# Circulating saturated fatty acids and risk of gestational diabetes mellitus: A cross-sectional study and meta-analysis

**DOI:** 10.3389/fnut.2022.903689

**Published:** 2022-08-01

**Authors:** Zhuo Sun, Zequn Deng, Xiaohui Wei, Na Wang, Jiaqi Yang, Wenyun Li, Min Wu, Yuwei Liu, Gengsheng He

**Affiliations:** ^1^School of Public Health, Key Laboratory of Public Health Safety, Ministry of Education, Fudan University, Shanghai, China; ^2^Nursing Department, Obstetrics and Gynaecology Hospital of Fudan University, Shanghai, China

**Keywords:** gestational diabetes mellitus, saturated fatty acids, cross-sectional study, meta-analysis, circulating fatty acids

## Abstract

**Background:**

Previous studies have analyzed the associations between the circulating saturated fatty acids (SFAs) and gestational diabetes mellitus (GDM), but no consistent conclusions have been reached. The aim of this study was to evaluate whether plasma SFAs were in correlation with GDM risks in our in-house women cross-sectional study and to better define their associations on the clinical evidence available to date by a dose-response meta-analysis.

**Methods:**

We carried out a cross-sectional study of 807 pregnant women in 2018–2019 (Shanghai, China). GDM was defined according to the criteria of the International Association of Diabetes and Pregnancy Study Groups (IADPSG). Gas chromatography was used to determine the plasma fatty acids (FAs) in the 24–28 gestational weeks. The SFAs levels of non-GDM and GDM participants were compared by Mann–Whitney test, and the association between SFAs and GDM was explored by multivariate logistic models. Further, the potential diagnostic value of plasma SFAs was evaluated using the method of receiver operating characteristic (ROC) analysis. For meta-analysis, five databases were systematically searched from inception to March 2022, and we included 25 relevant studies for calculating pooled standard mean differences (SMDs) and 95% CI to describe the differences in SFAs profiles between non-GDM and GDM women. Study-specific, multivariable-adjusted ORs and 95% CI were also pooled using a fixed-effect model or random-effects model according to the heterogeneity to evaluate the associations between circulating SFAs and GDM prevalence.

**Results:**

In our cross-sectional study, we found plasma proportion of palmitic acid (C16:0) was positively associated (aOR: 1.10 per 1% increase; 95% CI: 1.04, 1.17), while plasma stearic acid (C18:0) (aOR: 0.76 per 1% increase; 95% CI: 0.66, 0.89), arachidic acid (C20:0) (aOR: 0.92 per 0.1% increase; 95% CI: 0.87, 0.97), behenic acid (C22:0) (aOR: 0.94 per 0.1% increase; 95% CI: 0.92, 0.97), and lignoceric acid (C24:0) (aOR: 0.94 per 0.1% increase; 95% CI: 0.92, 0.97) were inversely associated with GDM. The area under the receiver operative characteristic curve increased from 0.7503 (the basic diagnostic model) to 0.8178 (*p* = 0.002) after adding total very-long-chain SFAs (VLcSFAs). A meta-analysis from 25 studies showed the circulating levels of three individual SFAs of GDM women were different from those of normal pregnant women. The summarized ORs for GDM was 1.593 (95% CI: 1.125, 2.255, *p* = 0.009), 0.652 (95% CI: 0.472, 0.901, *p* = 0.010) and 0.613 (95% CI: 0.449, 0.838, *p* = 0.002), respectively, comparing the highest vs. lowest quantile of the concentrations of C16:0, C22:0, and C24:0.

**Conclusion:**

Our results, combined with the findings from meta-analysis, showed that women with GDM had a particular circulating SFA profile, characterized by higher levels of palmitic acid, and lower levels of VLcSFAs. Alterations in the chain lengths of blood SFA profile were shown to be associated with the occurrence of GDM.

## Introduction

Gestational diabetes mellitus (GDM) refers to the glucose disorders that are first detected or occurs during pregnancy. It is one of the most common metabolic disorders during pregnancy. The prevalence of GDM varies from 6.1 to 15.2% in different regions worldwide (1). GDM causes adverse pregnancy outcomes, such as cesarean section rates, vascular pregnancy complications, macrosomia, and fetal hyperinsulinism. Early fetal exposure to an intrauterine high glucose environment can also lead to neurodevelopmental disorders, cognitive impairment, and an increased risk of obesity and cardiovascular disease later in life (2).

Nutritional status before and during pregnancy is an important factor for the occurrence of GDM. Saturated fatty acids (SFAs) are rich in foods such as meat, dairy products, and tropical vegetable oils. Several reports have shown SFAs having negative impacts on insulin sensitivity, leading to SFAs recommendations for the prevention and management of diabetes (3). However, no consistent conclusions have been reached on the association between SFAs and GDM (4–6). Numerous studies have described that the pregnant women with GDM had higher concentrations of total SFAs and long-chain saturated fatty acids (LcSFAs) than the controls (4), while there are still studies reporting negative findings (5, 6). The reasons for these inconsistencies might vary from small sample size, inconsistent sample type to different data collection methods.

Furthermore, contrary to the previous findings, recent studies have revealed a few SFAs that were somewhat beneficial to glucose control, such as odd chain fatty acids (OcSFAs) (7–9), very long-chain saturated fatty acids (VLcSFAs) (10, 11), and medium-chain fatty acids (McSFAs) (12, 13). Although the concentrations of these SFAs in human body are not as dominant as LcSFAs such as palmitic acid (C16:0) or stearic acid (C18:0), their contributions to the development of diabetes are worth discussing due to the increasing popularity of the foods sources as dairy products and coconut oil in China and worldwide (14, 15).

Do the blood types of SFAs affect the associations between food lipids and glycemic responses during pregnancy? To date, few studies have quantitatively investigated the associations between circulating SFAs and GDM, especially for the chain lengths of SFAs. Therefore, the objectives of this study were firstly to compare the circulating SFA profiles between GDM and non-GDM women, and to evaluate the effect of SFA types on the risk of developing GDM in our in-house cross-sectional study. Further, a meta-analysis of the evidences from both our study and a systematic literature search were conducted, with the dose-response relationship between circulating SFAs and the occurrence of GDM being explored.

## Materials and methods

### Cross-sectional study

#### Study design and population

This cross-sectional study was based on the data obtained from a longitudinal cohort conducted among pregnant women in the Obstetrics and Gynecology Hospital of Fudan University, Shanghai, China, to assess the association between maternal plasma FAs and GDM. The details of this cohort have been published previously (16). From October 2018 to March 2019, 1,008 pregnant women were recruited at 10–15 weeks of pregnancy according to the inclusion and exclusion criteria. After excluding participants with missing data (*n* = 119), failing to follow-up (*n* = 21), multiple births (*n* = 20), miscarriages (*n* = 19), stillbirth (*n* = 5), and pre-existing glucose intolerance (*n* = 17), a total of 807 pregnant women with a complete set of questionnaire data and plasma samples in the second trimester (24–28th weeks of gestation) were included for FAs analysis. The study was approved by the Research Committee of the Obstetrics and Gynecology Hospital of Fudan University (Ethics Approval number 2017-74), and written consents were obtained from all participants.

#### Diagnosis of gestational diabetes mellitus

At 24–28 weeks of gestation, after 8–12 h of fasting, pregnant women underwent a 75-g oral glucose tolerance test (OGTT). GDM was diagnosed according to the diagnostic criteria issued by the International Association of Diabetes and Pregnancy Study Groups (IADPSG) in 2010, which are defined by three cut-offs in different time points: fasting plasma glucose (FPG), OGTT-1h, or OGTT-2h plasma glucose (PG) concentrations ≥5.1, 10.0, or 8.5 mmol/L, respectively. Meeting or exceeding any of these three cut-offs was regarded as GDM (17).

#### Fatty acids measurements by gas chromatography

After an 8–12 h fast, blood samples were collected and aliquoted at the visits during the second trimester by trained nurses. The plasma concentrations of multiple FAs were measured by gas chromatography (GC, Shimadzu GC 2010, Shimadzu Corporation, Japan) with flame ionization detector (FID) analysis. Briefly, an internal standard, heptadecanoic acid (C17:0), was added to each sample, then total lipids were extracted from plasma by chloroform/methanol (2:1) using modified Folch procedure (18). FAs were derivatized using 14% boron trifluoride methanol and redissolved in n-hexane. The fatty acid (FA) separation was performed on an HP-88 column (100 m × 0.25 mm × 0.20 μm, Agilent Technologies, United States). The chromatographic peak height of each FA was measured using GCSolution software (Shimadzu Corporation, Japan).

In total, the absolute concentrations of 37 FAs, including saturated, monounsaturated, and polyunsaturated FAs, were measured. Total LcSFAs were the sum of SFA concentrations over 14 carbons. The total VLcSFAs concentration was the sum of the SFA concentrations over 20 carbons. The total FA concentration was the sum of the concentrations of all measured FAs.

#### Measurement of covariates

The basic information of the participants, including age, pre-pregnancy BMI, parity, cigarette smoking, alcohol drinking, recent physical activity, and first-degree family history of diabetes, was collected by questionnaires at recruitment. Then the age (in years) was divided into four categories: <25, 25–29, 30–34, and ≥35. Daily moderate-intensity physical activity (min/day) was divided into three categories: <10, 10–29, and ≥30. Cigarette smoking, alcohol drinking, and first-degree family history of diabetes were treated as dichotomized variables (yes/no/no clear). Parity was categorized as 0, 1, or ≥2. Pre-pregnancy BMI (kg/m^2^) was calculated by dividing pre-pregnancy weight in kilograms by the square of height in meters, and all the participants were classified as underweight (<18.5), normal weight (18.5–23.9), overweight (24.0–27.9), or obese (≥28.0), based on the BMI cut-offs for Chinese people (19). The nutritional status of pregnant women was obtained by two 3-day food records at 12–16 and 22–26 weeks of gestation. Participants were trained by clinical investigators to complete FRs and each 3-day record consisted of 2 weekdays and 1 weekend day. Specific quality control processes have been described in previous studies (16). We calculated daily food intake using the mean consumption of each type of food during the 6-day period and derived daily energy and nutrient intakes using the China Food Composition Database (20).

#### Measurement of metabolic biomarkers

Blood glucose measurements were performed by the electrochemical glucose oxidase method on an automatic biochemical analyzer (Hitachi 7600, Tokyo, Japan). Serum insulin was measured by radioimmunoassay (Johnson & Johnson kits). The homeostatic model assessment for insulin resistance (HOMA-IR) was estimated through the following formula: fasting glucose (mmol/L) × fasting insulin (mIU/L)/22.5 (21). TG, TC were measured using colorimetric methods (Wako Chemicals, Odawara, Japan) and HDL-C, LDL-C were measured using direct methods (Shanghai Beijia Biochemistry Reagents Co., Ltd., China) on an automatic biochemical analyzer (Hitachi 7600, Tokyo, Japan). All the measurements were performed by the biochemical laboratory of Obstetrics and Gynecology Hospital of Fudan University.

#### Statistical analysis

R 4.0.5 and Stata 16.0 (Stata Corp., College Station, TX, United States) were used for all analyses. A two-tailed *p*-value < 0.05 was regarded as statistically significant. Comparisons between groups were performed using χ^2^ tests for categorical variables and ANOVA or *t* tests for continuous variables with normal distribution. Mann–Whitney and Kruskal–Wallis tests were used for continuous variables with skewed distributions. Frequencies and percentages were used to describe the distributions of categorical variables. Continuous data were presented as means and standard deviations or medians (p50) or quartiles (p25 and p75). Pearson correlation was employed to explore the correlation between FAs and blood lipids.

Plasma FA percentages were reported as continuous variables or categorized into tertiles and considered as dependent variables. Univariate and multivariate logistic regression models were used to assess the relationships between FAs and the prevalence of GDM. The crude model did not adjust any other covariate. The adjusted model was adjusted for age, parity, cigarette smoking, alcohol drinking, physical activity, pre-pregnancy BMI, and first-degree family history of diabetes. All categorical covariates were coded into dummy variables. Test for linear trend was examined across tertiles of FAs by calculating the median value in each tertile and modeling it as a continuous variable. Odds ratios (ORs) and their 95% CI were estimated for the associations of individual FAs with prevalence of GDM through univariate (crude OR, cOR) and multivariable (adjusted OR, aOR) logistic regression models.

Further, receiver operating characteristic (ROC) analysis was used to identify whether plasma SFAs improved the diagnosis performance of conventional GDM risk factors. The basic diagnostic model only included FPG, TG (as continuous variables) and two other factors (as categorical variables) that were significantly different in univariate analysis: pre-pregnancy BMI and first-degree family history of diabetes. The area under the curve (AUC) of ROC was estimated in the basic model and the models with the addition of each FA (as a continuous variable).

### Meta-analysis

#### Registration of review protocol

The protocol for meta-analysis was registered in advance with PROSPERO database (International Prospective Register of Systematic Reviews; registration number: CRD42021277731^1^) before data extraction.

#### Study inclusion and exclusion strategies

Supplementary Text 1 showed the PICO strategy in this meta-analysis. The studies were included according to the following criteria: (1) observational studies, including cohort, nested case-control, case-control studies, and cross-sectional study; (2) patients with gestational diabetes identified in the clinical diagnosis according to the IADPSG criteria, Carpenter–Coustan (CC) criteria, the European Association for the Study of Diabetes (EASD) criteria, or other National Diabetes Data Group (NDDG) criteria (1, 22); (3) maternal blood were collected during pregnancy, including plasma, serum, or red blood cell (RBC); and (4) maternal SFAs, including myristic acid, palmitic acid, stearic acid, arachidic acid, behenic acid, and lignoceric acid, were measured by GC or GC/MS methods.

The exclusion criteria were: (1) patients did not meet the clinical diagnostic criteria for GDM, or had other types of pre-pregnancy diabetes or impaired glucose tolerance; (2) studies with insufficient or unextractable data; and (3) fatty acid measurements were performed on other samples rather than maternal blood samples.

#### Search strategy

The PubMed, Scopus, Web of Science, Chinese National Knowledge Information (CNKI), China Biology Medicine (CBM) databases were searched for relevant studies until October 2021, with no restriction of the initial date. An additional update of the search was performed in March 2022. The language of studies was restricted to Chinese and English only. Search terms included a range of Medical Subject Headings (MeSH) terms and their entry terms for gestational diabetes and FAs (Supplementary Text 2).

#### Data extraction

Endnote abstract format was exported from the results of each database search and screened by reading titles and abstracts by two researchers (ZS and XW) independently and discussed with a third researcher if disagreements arose. For eligible articles, the following information was extracted after reading the full text: first author’s name, publication year, type of study, the region where it was conducted, population characteristics (pre-pregnancy BMI classification, age, diagnostic criteria for cases, etc.), FA measurement methods, sampling time, and the number of participants. Where a single study reported multiple assessments of the same FA at different timepoints, all data were included in the meta-analysis. Where a single study reported multiple populations with different diagnostic criteria for GDM, the data of each population were separated for further analyses.

The means and standard deviations (SD) of FAs’ contents in each group were extracted, otherwise calculated from standard error (SE), range, or other convertible data. The ORs for the risk of gestational diabetes in the stratified group of FAs were also extracted.

#### Quality assessment

Two researchers (ZS and XW) independently completed quality assessments for each study according to the Newcastle-Ottawa Scale (NOS) for non-randomized studies (23). In this scale, a maximum of 9 points was assigned to each study, including 4 points for selection, 2 points for comparability, and 3 points for assessment of outcomes. Scores of 0–3, 4–6, and 7–9 were regarded as low, moderate, and high quality, respectively. The bias of publication was evaluated using funnel plot at contour strengthening and Egger’s test. Sensitivity analyses were carried out with the deletion of one study at a time to examine whether the pooled associations could be dramatically affected by a single study.

#### Statistical analysis

Pooled standard mean differences (SMDs) and confidence interval (95% CI) were calculated to describe the differences in FA profiles between women with and without GDM. The SMDs were calculated automatically by R package “meta” and estimated through the following formula: difference in outcome between groups/SD of outcome among participates (24).

Pooled ORs and 95% CI were calculated to better define the relationships between the types of circulating SFAs and GDM prevalence by comparing the FA’s highest stratification with the lowest stratification.

Heterogeneity between studies was evaluated by *I*^2^ statistics, whose value of 50% corresponded to the cut-off point for low and high degrees of heterogeneity. The fixed-effects model was implemented when the result of homogeneity *I*^2^ < 50%, otherwise using the random effects model.

For the dose-response analysis, studies that reported ≥3 FA quantiles were eligible. The median dose of each FA quantile and the related multivariable-adjusted OR and 95% CI were used. Linear dose-response analysis was first performed by using a generalized least-squares regression (GLS), followed by a non-linear Chi-square test. If the non-linear trend exhibited, a two-step random-effects dose-response model was used by modeling the dose using a restricted cubic spline model with three knots at 10, 50, and 90% of the distribution.

## Results

### Original cross-sectional study

#### Patient characteristics

Table 1 presented the sociodemographic, clinical and biochemical characteristics of the study participants, as specified by GDM status. Compared with the control subjects, the GDM patients were more likely to be overweight or obese before pregnancy (*p* = 0.003) and had a higher first-degree family history of diabetes (*p* = 0.008). FPG (GDM vs. non-GDM: 4.7 ± 0.6 vs. 4.2 ± 0.3, *p* < 0.001), OGTT-1h PG (GDM vs. non-GDM: 10.0 ± 1.3 vs. 6.9 ± 1.4, *p* < 0.001), OGTT-2h PG (GDM vs. non-GDM: 8.0 ± 1.4 vs. 5.9 ± 1.0, *p* < 0.001), insulin (GDM vs. non-GDM: 12.6 ± 7.3 vs. 9.0 ± 5.5, *p* < 0.001), HOMA-IR (GDM vs. non-GDM: 2.7 ± 1.9 vs. 1.7 ± 1.1, *p* < 0.001), and TG (GDM vs. non-GDM: 2.7 ± 0.9 vs. 2.4 ± 0.9, *p* = 0.001) in GDM patients were higher than those in the control. Table 1 also showed the energy and macronutrient intakes of the study participants. The daily energy intake of the participants reached an average of 2,126.4 ± 695.5 kcal/day, and there were no significant differences between GDM and non-GDM pregnant women in the energy, carbohydrate, protein, and fat intakes.

**TABLE 1:** The basic characteristic of the participants in the cross-sectional study by GDM status.

	Non-GDM	GDM	Total	*p*-Value*
	*N* (%)	*N* (%)	*N* (%)	
All	746	61	807	
Age, year				0.182
<25	18 (2.4)	0 (0)	18 (2.2)	
25–29	280 (37.5)	16 (26.2)	296 (36.7)	
30–34	357 (47.9)	36 (59.0)	393 (48.7)	
≥35	91 (12.2)	9 (14.8)	100 (12.4)	
BMI				0.003
<18.5	126 (16.9)	5 (8.2)	131 (16.2)	
18.5–23.9	533 (71.4)	39 (63.9)	572 (70.9)	
24.0–27.9	71 (9.5)	13 (21.3)	84 (10.4)	
≥28	16 (2.1)	4 (6.6)	20 (2.5)	
Parity				0.351
1	598 (80.2)	53 (86.9)	651 (80.7)	
2	146 (19.6)	8 (13.1)	154 (19.1)	
≥3	2 (0.3)	0 (0)	2 (0.2)	
Cigarette smoking				1.000
No	726 (97.3)	60 (98.4)	786 (97.4)	
Yes	20 (2.7)	1 (1.6)	21 (2.6)	
Alcohol drinking				0.500
No	677 (90.8)	54 (88.5)	731 (90.6)	
Yes	69 (9.2)	7 (11.5)	76 (9.4)	
Moderate-intensity physical activity, min/day				0.839
<10	705 (94.5%)	59 (96.7%)	764 (94.7%)	
10–29	36 (4.8%)	2 (3.3%)	38 (4.7%)	
≥30	5 (0.6%)	0 (0%)	5 (0.6%)	
First-degree family history of diabetes				0.008
Yes	79 (10.6)	15 (24.6)	94 (11.7)	
No	664 (89.0)	46 (75.4)	710 (88.0)	
Not clear	3 (0.4)	0 (0)	3 (0.4)	
	**Mean ± SD**	**Mean ± SD**	**Mean ± SD**	
FPG, mmol/L	4.2 ± 0.3	4.7 ± 0.6	4.3 ± 0.4	<0.001
OGTT-1h PG, mmol/L	6.9 ± 1.4	10.0 ± 1.3	7.1 ± 1.6	<0.001
OGTT-2h PG, mmol/L	5.9 ± 1.0	8.0 ± 1.4	6.0 ± 1.2	<0.001
Insulin, μIU/mL	9.0 ± 5.56.	12.6 ± 7.3	9.2 ± 5.8	<0.001
HOMA-IR	1.7 ± 1.1	2.7 ± 1.9	1.8 ± 1.2	<0.001
TG, mmol/L	2.4 ± 0.9	2.7 ± 0.9	2.4 ± 0.9	0.001
TC, mmol/L	6.1 ± 1.0	6.2 ± 0.8	6.1 ± 1.0	0.680
HDL-C, mmol/L	1.5 ± 0.5	1.5 ± 0.4	1.5 ± 0.5	0.890
LDL-C, mmol/L	3.8 ± 1.2	3.9 ± 1.0	3.8 ± 1.2	0.784
Carbohydrate intake, g/day	236.0 ± 116.1	226.4 ± 197.6	235.3 ± 115.8	0.535
Fat intake, g/day	81.5 ± 30.4	85.1 ± 24.5	81.8 ± 30.0	0.368
Protein intake, g/day	88.6 ± 47.2	91.2 ± 46.7	88.8 ± 47.1	0.679
Total energy intake, kcal/day	2123.7 ± 706.3	2159.3 ± 550.6	2126.4 ± 695.5	0.701

*The data of GDM and non-GDM pregnant women were compared. Comparisons between groups were performed using χ^2^ tests for categorical variables and *t* tests for continuous variables with normal distribution.

Table 2 showed the plasma SFA profiles (% of total FAs) of all participants, as specified by GDM status. The concentrations of plasma SFAs were shown in Supplementary Table 1. Participants with GDM were more likely to have a lower percentage of VLcSFAs [GDM vs. non-GDM: 0.24 (0.21, 0.29) vs. 0.66 (0.24, 1.73), *p* < 0.001] and a higher percentage of palmitic acid [C16:0, GDM vs. non-GDM: 29.7 (28.0, 31.5) vs. 27.1 (24.4, 30.1), *p* < 0.001]. Total FAs, total SFAs, and LcSFAs did not differ significantly between GDM or Non-GDM participants.

**TABLE 2:** Plasma SFAs (percentage, % of total fatty acids) in pregnant women with vs. without GDM in the second trimesters.

	Non-GDM	GDM	Total	*p*-Value*
	Median (P25, P75)	Median (P25, P75)	Median (P25, P75)	
Myristic acid (C14:0)	0.86 (0.65, 1.25)	0.86 (0.66, 1.07)	0.86 (0.65, 1.21)	0.288
Palmitic acid (C16:0)	27.1 (24.4, 30.1)	29.7 (28.0, 31.5)	27.3 (24.5, 30.3)	<0.001
Stearic acid (C18:0)	5.92 (4.83, 7.25)	4.71 (3.84, 5.77)	5.85 (4.71, 7.18)	<0.001
Arachidic acid (C20:0)	0.08 (0.04, 0.15)	0.05 (0.03, 0.08)	0.08 (0.04, 0.14)	<0.001
Behenic acid (C22:0)	0.16 (0.03, 0.29)	0.03 (0.02, 0.05)	0.14 (0.02, 0.29)	<0.001
Lignoceric acid (C24:0)	0.08 (0.07, 0.13)	0.07 (0.06, 0.08)	0.08 (0.07, 0.12)	<0.001
VLcSFAs	0.66 (0.24, 1.73)	0.24 (0.21, 0.29)	0.57 (0.23, 1.64)	<0.001
LcSFAs	35.4 (31.8, 40.2)	36.2 (33.0, 38.5)	35.4 (31.9, 39.9)	0.865
Total SFAs	40.7 (36.9, 44.8)	42.1 (39.7, 44.5)	40.9 (37.1, 44.8)	0.128

*The data of GDM and non-GDM pregnant women were compared by Mann–Whitney tests.

#### Correlations between circulating saturated fatty acids and blood lipids

Table 3 shows the correlations between various SFAs and blood lipids. Total SFAs was strongly positively correlated with total LcSFAs (Pearson correlation coefficient: 0.94), palmitic acid (C16:0) (Pearson correlation coefficient: 0.93), and a weak correlation was found with VLcSFAs (Pearson correlation coefficient: 0.39). TG was moderately correlated with total SFAs (Pearson correlation coefficient: 0.24), LcSFAs (Pearson correlation coefficient: 0.26), and palmitic acid (C16:0) (Pearson correlation coefficient: 0.32), but not with the other FA variables. Moreover, there was significant collinearity among total SFAs, LcSFAs, and palmitic acid (C16:0). No significant collinearity was found between TG and all FA variables.

**TABLE 3:** The correlation of various SFAs in the cross-sectional study.

	C14:0	C16:0	C18:0	C20:0	C22:0	C24:0	VLcSFAs	LcSFAs	Total SFAs
C14:0	1.00								
C16:0	0.04	1.00							
C18:0	0.06	0.89*	1.00						
C20:0	0.06	0.66*	0.78*	1.00					
C22:0	0.16*	0.59*	0.69*	0.65*	1.00				
C24:0	0.03	0.64*	0.63*	0.60*	0.48*	1.00			
VLcSFAs	0.01	0.12*	0.12*	0.11*	0.10*	0.10*	1.00		
LcSFAs	0.20*	0.98*	0.93*	0.71*	0.65*	0.65*	0.12*	1.00	
Total SFAs	0.15*	0.93*	0.86*	0.67*	0.58*	0.61*	0.39*	0.94*	1.00
TG	0.02	0.32*	0.09*	0.03	0.05	0.06	0.01	0.26*	0.24*
TC	0.05	0.07	0.05	−0.01	0.01	−0.02	−0.01	0.07*	0.05
HDL-C	0.05	0.10*	0.10*	0.03	0.06	0.02	0.01	0.10*	0.08*
LDL-C	0.07*	0.06	0.06	0.01	0.04	0.01	0.02	0.07*	0.05*

All FAs were presented as absolute concentrations and Pearson correlation coefficient were showed.

*p < 0.05.

#### Associations between plasma fatty acid profile and gestational diabetes mellitus

Adjusted ORs and 95% CIs estimated according to the tertiles of plasma SFAs and GDM risks were shown in Table 4. Based on the adjusted models, SFAs were differently associated with GDM in opposing directions. Compared with the first tertiles, plasma palmitic acid (C16:0, *p* trend = 0.002) was positively associated, while plasma stearic acid (C18:0, *p* trend <0.001), arachidic acid (C20:0, *p* trend <0.001), behenic acid (C22:0, *p* trend <0.001), and lignoceric acid (C24:0, *p* trend <0.001) were inversely associated with GDM. Meanwhile, plasma total SFAs showed no significant correlation with GDM. When FAs were included in the model as a continuous variable, after adjusting TG level and other covariates, plasma concentration of palmitic acid (C16:0) was positively associated (aOR: 1.10 per 1% increase; 95% CI: 1.04, 1.17), while plasma stearic acid (C18:0) (aOR: 0.76 per 1% increase; 95% CI: 0.66, 0.89), arachidic acid (C20:0) (aOR: 0.92 per 0.1% increase; 95% CI: 0.87, 0.97), behenic acid (C22:0) (aOR: 0.94 per 0.1% increase; 95% CI: 0.92, 0.97), and lignoceric acid (C24:0) (aOR: 0.94 per 0.1% increase; 95% CI: 0.92, 0.97) were inversely associated with GDM.

**TABLE 4:** Crude and adjusted ORs (95% CIs) for risk of GDM according to tertiles of plasma SFAs in the second trimesters in the cross-sectional study.

	Tertiles of specific SFA			As continuous variable	*p* for trend*
	Q1	Q2	Q3		
**Myristic acid (C14:0)**					
Case/control	21/248	25/244	15/254		
Crude model	1.00 (reference)	1.21 (0.66, 2.22)	0.70 (0.35, 1.38)	0.70 (0.47, 1.04)	0.220
Adjusted model 1	1.00 (reference)	1.26 (0.68, 2.32)	0.72 (0.36, 1.45)	0.70 (0.47, 1.05)	0.262
Adjusted model 2	1.00 (reference)	1.11 (0.59, 2.06)	0.66 (0.33, 1.34)	0.70 (0.47, 1.05)	0.195
Adjusted model 3	1.00 (reference)	1.10 (0.59, 2.05)	0.66 (0.33, 1.34)	0.68 (0.44, 1.05)	0.192
**Palmitic acid (C16:0)**					
Case/control	8/261	19/250	34/235		
Crude model	1.00 (reference)	2.48 (1.07, 5.77)	4.72 (2.14, 10.40)	1.10 (1.05, 1.17)	< 0.001
Adjusted model 1	1.00 (reference)	2.34 (1.00, 5.47)	4.38 (1.97, 9.72)	1.10 (1.04, 1.16)	< 0.001
Adjusted model 2	1.00 (reference)	2.12 (0.90, 4.99)	4.08 (1.82, 9.13)	1.10 (1.04, 1.17)	0.001
Adjusted model 3	1.00 (reference)	2.13 (0.90, 5.01)	4.10 (1.83, 9.17)	1.10 (1.04, 1.17)	0.002
**Stearic acid (C18:0)**					
Case/control	33/236	20/249	8/261		
Crude model	1.00 (reference)	0.57 (0.32, 1.03)	0.22 (0.10, 0.48)	0.79 (0.69, 0.91)	< 0.001
Adjusted model 1	1.00 (reference)	0.54 (0.30, 0.98)	0.22 (0.10, 0.49)	0.80 (0.70, 0.92)	< 0.001
Adjusted model 2	1.00 (reference)	0.49 (0.27, 0.90)	0.21 (0.09, 0.46)	0.77 (0.66, 0.89)	< 0.001
Adjusted model 3	1.00 (reference)	0.49 (0.27, 0.90)	0.21 (0.09, 0.46)	0.76 (0.66, 0.89)	< 0.001
**Arachidic acid (C20:0)**					
Case/control	31/238	24/245	6/263		
Crude model	1.00 (reference)	0.75 (0.43, 1.32)	0.18 (0.07, 0.43)	0.92 (0.87, 0.96)	< 0.001
Adjusted model 1	1.00 (reference)	0.72 (0.41, 1.27)	0.19 (0.08, 0.45)	0.92 (0.87, 0.97)	< 0.001
Adjusted model 2	1.00 (reference)	0.63 (0.35, 1.13)	0.18 (0.07, 0.44)	0.92 (0.87, 0.97)	< 0.001
Adjusted model 3	1.00 (reference)	0.63 (0.35, 1.14)	0.18 (0.07, 0.45)	0.92 (0.87, 0.97)	< 0.001
**Behenic acid (C22:0**)					
Case/control	42/228	12/254	7/264		
Crude model	1.00 (reference)	0.25 (0.13, 0.49)	0.14 (0.06, 0.33)	0.94 (0.92, 0.97)	< 0.001
Adjusted model 1	1.00 (reference)	0.26 (0.13, 0.51)	0.15 (0.06, 0.34)	0.95 (0.92, 0.97)	< 0.001
Adjusted model 2	1.00 (reference)	0.23 (0.12, 0.45)	0.13 (0.06, 0.31)	0.94 (0.92, 0.97)	< 0.001
Adjusted model 3	1.00 (reference)	0.23 (0.12, 0.46)	0.13 (0.06, 0.31)	0.94 (0.92, 0.97)	< 0.001
**Lignoceric acid (C24:0)**					
Case/control	28/241	27/242	6/263		
Crude model	1.00 (reference)	0.96 (0.55, 1.68)	0.20 (0.08, 0.48)	0.90 (0.84, 0.97)	< 0.001
Adjusted model 1	1.00 (reference)	1.02 (0.58, 1.80)	0.21 (0.08, 0.51)	0.91 (0.85, 0.97)	< 0.001
Adjusted model 2	1.00 (reference)	0.92 (0.52, 1.63)	0.20 (0.08, 0.49)	0.90 (0.83, 0.97)	< 0.001
Adjusted model 3	1.00 (reference)	0.95 (0.53, 1.69)	0.20 (0.06, 0.50)	0.90 (0.83, 0.97)	< 0.001
**VLcSFAs**					
Case/control	40/229	15/254	6/263		
Crude model	1.00 (reference)	0.34 (0.18, 0.63)	0.13 (0.05, 0.31)	0.97 (0.95, 0.98)	< 0.001
Adjusted model 1	1.00 (reference)	0.35 (0.19, 0.65)	0.14 (0.06, 0.34)	0.97 (0.95, 0.98)	< 0.001
Adjusted model 2	1.00 (reference)	0.30 (0.16, 0.58)	0.13 (0.05, 0.32)	0.96 (0.95, 0.98)	< 0.001
Adjusted model 3	1.00 (reference)	0.31 (0.16, 0.58)	0.13 (0.05, 0.32)	0.96 (0.95, 0.98)	< 0.001
**LcSFAs**					
Case/control	16/253	28/241	17/252		
Crude model	1.00 (reference)	1.84 (0.97, 3.48)	1.07 (0.53, 2.16)	1.00 (0.97, 1.04)	0.907
Adjusted model 1	1.00 (reference)	1.81 (0.95, 3.44)	1.06 (0.52, 2.16)	1.00 (0.96, 1.04)	0.924
Adjusted model 2	1.00 (reference)	1.66 (0.86, 3.19)	1.06 (0.49, 2.07)	0.99 (0.95, 1.03)	0.960
Adjusted model 3	1.00 (reference)	1.66 (0.86, 3.19)	1.01 (0.49, 2.08)	0.99 (0.95, 1.03)	0.975
**Total SFAs**					
Case/control	10/259	30/239	21/248		
Crude model	1.00 (reference)	3.25 (1.56, 6.79)	2.19 (1.01, 4.75)	1.02 (0.98, 1.06)	0.267
Adjusted model 1	1.00 (reference)	3.16 (1.50, 6.64)	2.23 (1.02, 4.87)	1.02 (0.98, 1.06)	0.259
Adjusted model 2	1.00 (reference)	2.99 (1.41, 6.35)	2.22 (1.00, 4.90)	1.02 (0.98, 1.06)	0.371
Adjusted model 3	1.00 (reference)	3.08 (1.44, 6.56)	2.33 (1.05, 5.19)	1.02 (0.98, 1.07)	0.294

*Test for trend based on the variable containing the median value for each tertile. ORs and 95% CIs were calculated with the use of logistic regression models. Crude model did not adjust any covariant; covariates in the adjusted model 1 included per-pregnancy BMI and age. Covariates in the adjusted model 2 included those in adjusted model 1 and parity, cigarette smoking, alcohol drinking, recent physical activity, and first-degree family history of diabetes. Covariates in the adjusted model 3 included those in adjusted model 2 and TG level. ORs (95% CIs) for GDM and FAs (as continuous variable) represented each 1% increase in circulating C14:0–C18:0, LcSFAs, SFAs or 0.1% increase in circulating C20:0–C:24:0, VLcSFAs.

**FIGURE 1:**
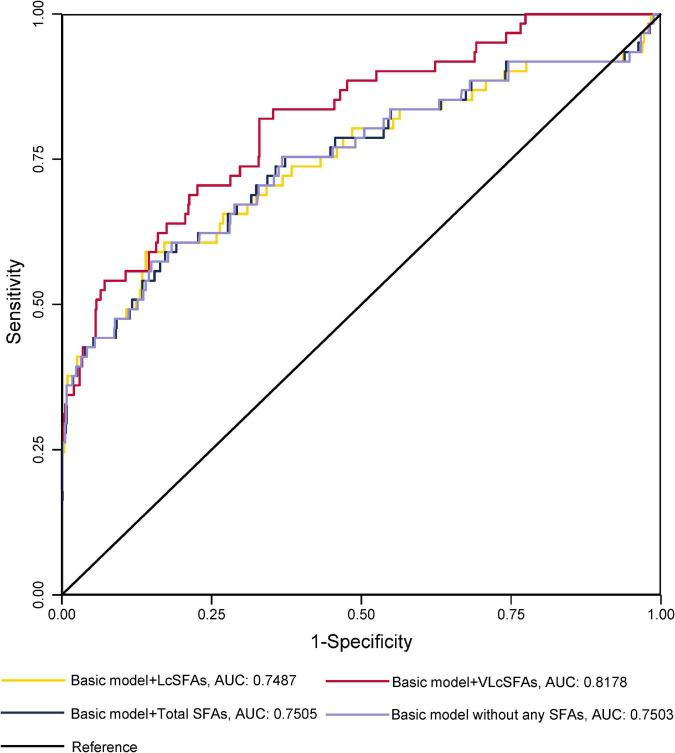
Receiver operating characteristic curves without or with the addition of a SFA category for GDM diagnostic models in cross-sectional study participants.

**FIGURE 2:**
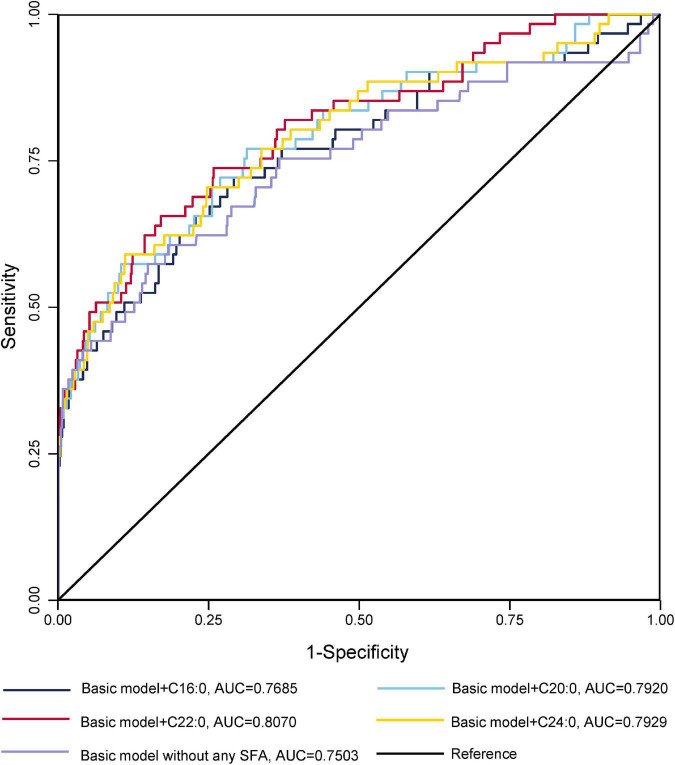
Receiver operating characteristic curves for GDM without or with the addition of a single SFA for GDM diagnostic models in cross-sectional study participants.

The ROC curves for GDM diagnostic model were shown in Figures 1, 2. Figure 1 demonstrated four models of summarized FAs. Adding VLcSFAs to the basic model achieved the largest AUC of 0.8178 and was the only model that differed significantly from the basic model (AUC = 0.7503, *p* = 0.002). Among the models of single FA in Figure 2, AUC of the basic model with behenic acid (C22:0) was the largest (AUC = 0.8070), which significantly differed from the basic model (AUC = 0.7503, *p* = 0.033). However, the models with stearic acid (C18:0) did not differ significantly from the basic model.

### Meta-analysis

#### Literature search and study characteristics of meta

A total of 25 studies with 7,782 individuals were analyzed in the meta-analysis, including our current original cross-sectional study. A PRISMA flow diagram was shown in Supplementary Table 1.

Supplementary Table 2 summarized the characteristics of included studies, including 4 cohort studies, 13 case-control studies, 7 nested case-control studies, and 1 cross-sectional study. Among which, 11 studies were from Europe, 8 were from Asia, 6 were from the North America; 22 studies reported the blood proportions of FAs and 11 studies reported the concentrations of FAs. Two studies reported data at three time points of the early, middle, and late pregnancy, and three studies reported data at two time points. Three studies reported data from more than one case group, categorizing cases by BMI, diagnostic criteria, or whether they received insulin therapy.

Six studies involving 1,233 cases among 3,139 individuals reported the ORs of SFAs on GDM prevalence (Supplementary Table 3), in which all the studies analyzed myristic acid (C14:0), palmitic acid (C16:0), and stearic acid (C18:0), 5 studies reported lignoceric acid (C24:0) and 4 reported arachidic acid (C20:0), and behenic acid (22:0).

#### Risk-of-bias assessment and study quality of meta

Overall, NOS scores ranged from 6 to 9 among selected studies, with the data of 18 studies scoring ≥8 (Supplementary Table 5). In terms of selection bias and comparability, nine studies did not state the medical history of the control group and eight studies did not mention the matching method for controls.

#### Meta-analysis for mean differences of maternal circulating saturated fatty acid profiles

**FIGURE 3:**
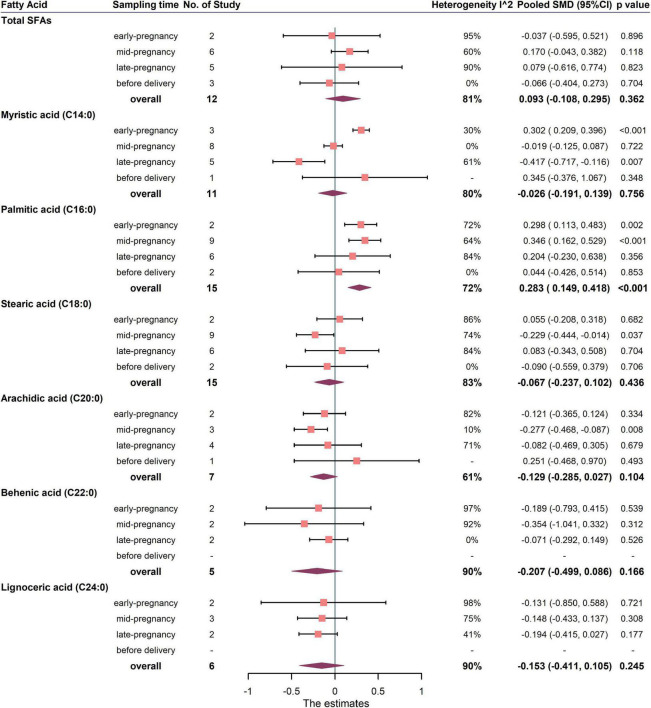
Summary forest plot of pooled SMDs for total SFAs and each SFA as percentage of total fatty acids in pregnant women with and without GDM. Dots and horizontal lines represent SMDs and 95% CIs. Diamonds depict pooled estimates. The forest plots of the original studies were shown in the Supplementary Figure 2.

**FIGURE 4:**
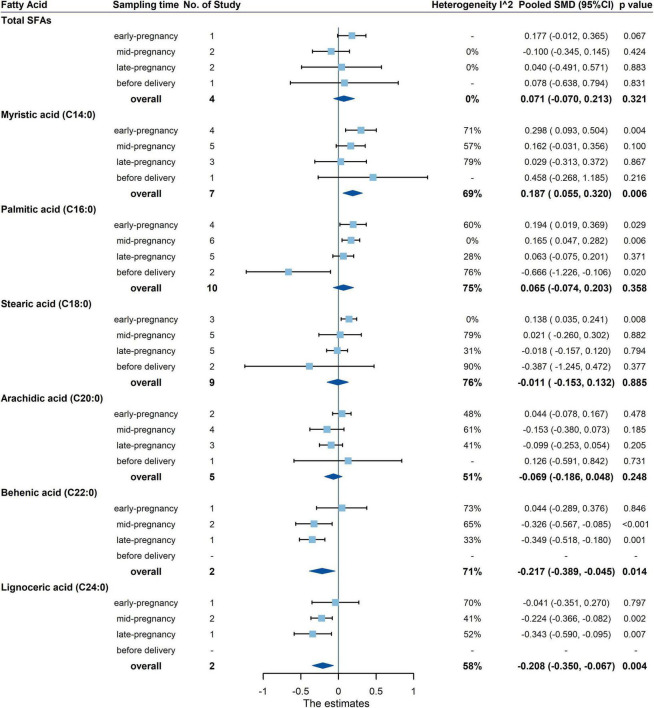
Summary forest plot of pooled SMDs for total SFAs and each SFA as concentration in pregnant women with and without GDM. Dots and horizontal lines represent SMDs and 95% CIs. Diamonds depict pooled estimates. The forest plots of the original studies were shown in the Supplementary Figure 3.

The primary outcomes for the maternal total SFAs between the GDM women and the healthy controls were shown in Figures 3, 4. The results showed no difference in the percentages or concentrations of total SFAs between two groups (percentage: 12 studies, pooled SMD: 0.093, 95% CI: −0.108, 0.295, *p* = 0.362; concentration: 4 studies, pooled SMD: 0.071, 95% CI: −0.070, 0.213, *p* = 0.321).

Summary forest plot of the pooled SMDs for individual SFA in cases and controls was shown in Figure 3, 4 (as detailed in Supplementary Figures 2, 3). In terms of percentage, the blood level of palmitic acid (C16:0) was higher in pregnant women with GDM than in the control group (15 studies, pooled SMD: 0.283, 95% CI: 0.149, 0.418, *p* < 0.001). For trimester subgroups, circulating palmitic acid (C16:0) increased significantly in the early and second trimesters of pregnancy, but not in the third trimester.

In terms of concentration, the level of myristic acid (C14:0) was higher in pregnant women with GDM than in the control group (7 studies, pooled SMD: 0.187, 95% CI: 0.055, 0.320, *p* = 0.006), while the levels of behenic (C22:0) and lignoceric acid (C24:0) were lower in GDM. For trimester subgroups, circulating arachidic acid (C20:0) and behenic acid (C22:0) concentrations in GDM women decreased significantly in the second and third trimesters.

To further assess the sources of potential bias, subgroup analysis was carried out according to the stated factors in Supplementary Table 4. Except for the RBC subgroup, the percentages of palmitic acid (C16:0) were found higher in the women with GDM in other subgroups. Meta-analysis for the associations between circulating SFAs and GDM prevalence.

**FIGURE 5:**
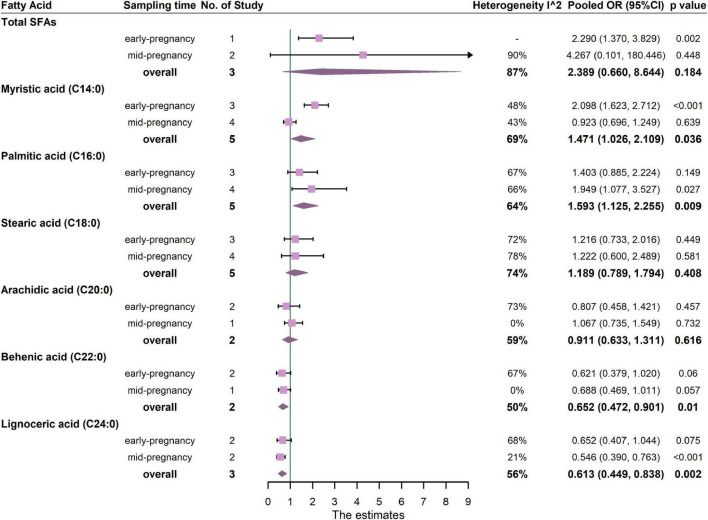
Summary forest plot of pooled ORs of GDM for total SFAs and each SFA. Dots and horizontal lines represent ORs and 95% CIs. For each study, OR corresponds to the comparison of extreme quantiles of each saturated fatty acid. Diamonds depict pooled estimates from random-effects inverse-variance–weighted meta-analyses. Subgroup analysis was performed according to the sampling time of biological samples. The forest plots of the original studies were shown in the Supplementary Figure 4.

The associations between maternal total SFAs and GDM were shown in Figure 5. The results from three studies showed that there was no significant association between total SFAs and the risk of GDM (pooled OR: 2.389, 95% CI: 0.660, 8.644, *p* = 0.184, *I*^2^ = 87%). One study measured SFAs in the first trimester and two studies measured SFAs in the second trimester. For trimester subgroups, circulating SFAs in early pregnancy were positively correlated with the occurrence of GDM, but not in second trimester (Figure 5).

In the analysis of FA subtypes (Figure 5), palmitic acid (C16:0) was positively associated with GDM (5 studies, OR: 1.593, 95% CI: 1.125, 2.255, *p* = 0.009, *I*^2^ = 64%), whereas both behenic acid and lignoceric acid (C22:0, OR: 0.652, 95% CI: 0.472, 0.901, *p* = 0.010, *I*^2^ = 50%; C24:0, OR: 0.613, 95% CI: 0.449, 0.838, *p* = 0.002, *I*^2^ = 56%, respectively) were inversely associated with GDM. For trimester subgroups, palmitic acid (C16:0) was positively associated with GDM in the second trimester of pregnancy but not in the early. In addition, lignoceric acid (C24:0) was negatively associated with GDM in the second trimester.

**FIGURE 6:**
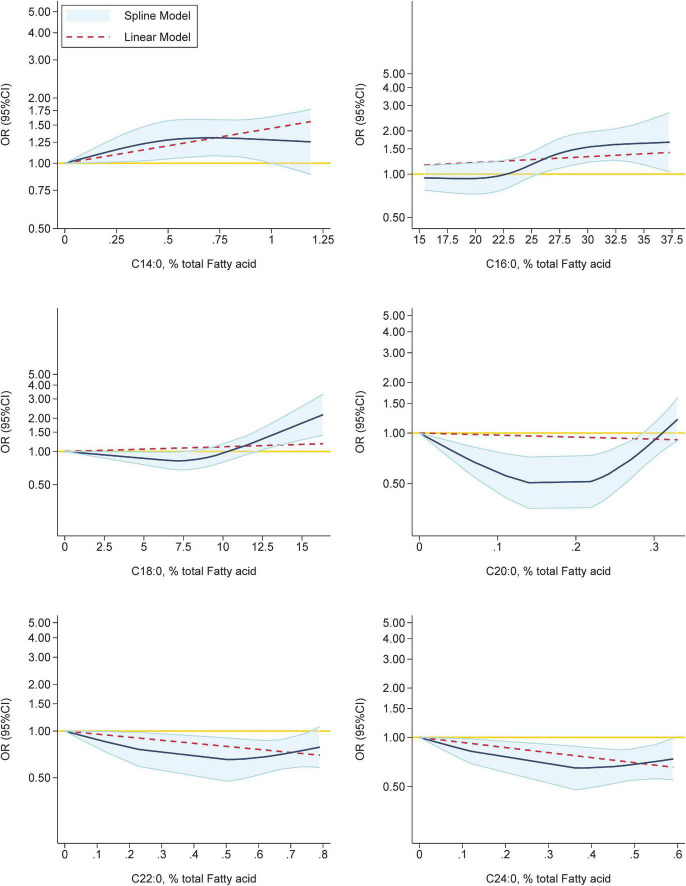
Dose-response meta-analysis for associations between SFAs and the prevalence of GDM based on data from prospective studies. The pooled OR trends by SFAs’ percentage (solid navy-blue lines) and their 95% CIs (light-blue areas) were obtained by random-effects dose–response meta-analysis. The dashed red lines represent the linear trend. The circles represent ORs according to the specific fatty acid’s categories from each study. The yellow line was the horizontal reference line (*Y* = 1.00).

As shown in Figure 6, when palmitic acid (C16:0, *p*-linearity < 0.003; *p*-curvilinearity < 0.003) or stearic acid (C18:0, *p*-linearity = 0.258; *p*-curvilinearity = 0.0006) accounted for more than 26 and 13.5% of total FAs respectively, the risk of GDM increased (OR >1). The non-linear dose-response relationships for the remaining FAs all exhibited non-monotonic trends, partly due to the small number of studies reported. Significant linear and curvilinear associations with GDM were found for myristic acid (C14:0, *p*-linearity = 0.011; *p*-curvilinearity = 0.034), behenic acid (C22:0, *p*-linearity = 0.016; *p*-curvilinearity = 0.025) and lignoceric acid (C24:0, *p*-linearity = 0.005; *p*-curvilinearity = 0.008). Curvilinear associations with GDM, rather than linear associations, were found for arachidic acid (C20:0, *p*-linearity = 0.197; *p*-curvilinearity < 0.001).

#### Sensitivity analysis and publication bias of meta

The results of sensitivity analysis were shown in Supplementary Figures 5–7. Most of the results remained unchanged after removing one study, so the conclusions were considered robust.

Funnel plots of publication bias were presented in Supplementary Figures 8, 9. Among the continuous data of circulating SFA levels in GDM pregnant women and the control group, no publication bias was found. Among the ORs of circulating SFA, the data of palmitic acid (C16:0) (Supplementary Figures 9B, Egger’s test was adopted and *p* = 0.0013) had publication bias, but sensitivity analysis showed that removing any one data set did not affect the results for palmitic acid (C16:0, Supplementary Figure 7C). The ORs of total SFAs also had publication bias (Egger’s test was adopted and *p* = 0.0444), and the reason might be the limited number of studies.

## Discussion

Circulating FA levels have been adopted in recent years to represent the fat intakes, especially for essential FAs and their derivatives. In the study of our in-house cross-sectional study, as well as the meta-analysis, we found that the women with gestational diabetes had a distinct circulating SFA profile, characterized by higher levels of palmitic acid (C16:0), and lower levels of VLcSFAs (C20:0, C22:0, and C24:0).

As for palmitic acid (C16:0), our original cross-sectional study showed that both the plasma percentage and concentration were higher in GDM women in the second trimester, and were positively associated with the prevalence of GDM. Similarly, the meta results clearly demonstrated that circulating palmitic acid had the strongest positive correlation with GDM, both in the early and second trimester of pregnancy. These results were partially in agreement with many individual studies included in our meta-analysis, such as the 1:2 case-control study conducted by (4) and the nested case-control study conducted by (25), which indicated that the circulating palmitic acid (C16:0) proportion of pregnant women with GDM was higher than that of the control. However, there were also studies showing no differences in RBC C16:0 ratios between the pregnant women with or without GDM. And it was worth noticing that this population had relatively low C16:0 levels, ranging from 16 to 17% (26). Did the absolute concentration or proportion of palmitic acid modify their relations to GDM? A further dose-response analysis between the palmitic acid proportion and GDM in this study showed a monotonic increase trend. When the blood proportion of palmitic acid exceeded 26%, the occurrence of GDM began to increase. Our findings could be partially explained by the commonly believed disturbing effects of palmitic acid on insulin sensitivity: (1) activated pro-inflammatory signaling pathways and/or through the synthesis of diacylglycerol and ceramide (27, 28); (2) activated Toll-Like Receptor 4 (TLR4) in white adipose tissue and immune cells and stimulates the downstream pro-inflammatory process (27–30); (3) induced endoplasmic reticulum stress in immune cells, thereby activating NF-κB signaling pathway and inflammatory cascades (27, 28, 31).

Additionally, our cross-sectional data demonstrated that the levels of circulating VLcSFAs [arachidic acid (C20:0), behenic acid (C22:0), and lignoceric acid(C24:0)] in GDM women during the second trimester of pregnancy were significantly lower than non-GDM women, and adding circulating VLcSFA levels could increase the accuracy of GDM diagnosing in the second trimester. In addition, our meta results also suggested those higher levels of circulating VLcSFAs were associated with a lowered risk of GDM, and the chain length of VLcSFA might be a key factor. To our knowledge, this was the first meta-analysis examining the associations between circulating VLcSFAs and the risk of GDM. VLcSFAs has been reported to be inversely associated with multiple metabolic outcomes (10, 32, 33). Our result was consistent with a previous meta-study of type 2 diabetes by pooling data from 12 prospective studies (34) and another meta-analysis study of metabolic syndrome (35). The biological theory to the protective effects of VLcSFAs on diabetes has been inconclusive, but several mechanisms have been proposed. On one hand, the chain length of FAs might modulate the effect of ceramide on insulin sensitivity in the liver and surrounding tissues (36). Ceramide containing LcSFA such as C16:0 could increase insulin resistance (36), while the ceramides containing VLcSFAs were negatively correlated with hepatic insulin resistance (37, 38). On the other hand, VLcSFAs could interact with peroxisome proliferator-activated receptor (PPAR) δ, which was opposed to the other FAs with PPARα (39). PPARδ activation in the small intestine might potentiate the production of glucagon-like peptide (GLP)-1, which preserved β cell morphology and function, thereby increasing systemic insulin sensitivity (40). Collectively, these findings on chain lengths suggested that the levels of VLcSFAs might be potential biomarkers for predicting the occurrence of GDM.

Previous systematic reviews and meta-studies have explored the relationship between circulating FAs, especially for polyunsaturated FAs, and GDM, but no conclusion has been reached for SFAs (41, 42). In our original cross-sectional study, although the levels of plasma total SFAs were moderately higher in pregnant women with GDM than in controls, they were not statistically significant and were also not associated with the risk of GDM. The meta results of SMDs and ORs also showed that there was no significant association between total SFAs and the risk of GDM. As distinct associations were detected between a particular SFA and GDM, the constitution of FAs pool would greatly affect the overall associations.

Several limitations of this study needed to be considered when interpreting the results. Firstly, our original study was a cross-sectional study, making it difficult to explore the temporality and causality of associations. Secondly, since many studies provided the ratio of SFAs to total FAs rather than absolute concentration, the intercorrelations of SFAs made it challenging to explain the independent association between single SFA and diabetes risk. In addition, although we included data from 24 studies, not all studies reported VLcSFAs, which might lead to partial publication bias. Still, our study had several strengths. To our knowledge, this was the most comprehensive analysis to date to examine the associations of circulating VLcSFAs with GDM, with the dose-response relationship between SFAs and GDM explored. Our original cross-sectional study highlighted the association of plasma VLcSFAs level with the occurrence of GDM and found that VLcSFAs may be a potential biomarker for GDM by ROC analysis. Moreover, the studies we included in meta-analysis were generally of high quality, with unified and accurate FA measurement methods.

## Conclusion

Our results, combined with the findings from meta-analysis, showed that women with GDM had a particular circulating SFA profile, manifested by higher levels of palmitic acid, and lower levels of VLcSFAs. Alterations in the blood SFA profile, especially the proportion of SFAs with different chain lengths, might be associated with the onset of gestational diabetes. Further studies are needed to clarify the long-term effects of VLcSFAs on the postnatal outcomes of GDM mothers and birth outcomes of their fetuses.

## Data availability statement

The raw data supporting the conclusions of this article will be made available by the authors, without undue reservation.

## Ethics statement

The studies involving human participants were reviewed and approved by the Ethics Committee of the Obstetrics and Gynecology Hospital of Fudan University (Ethics Approval number 2017-74). The patients/participants provided their written informed consent to participate in this study.

## Author contributions

GH and YL conceptualized the study idea and methodological approach, provided advice on interpreting the results, supervised data collection, and edited the drafts. ZS carried out the laboratory testing, data collection, statistical analyses, and prepared original draft preparation. ZD and NW carried out field investigation, laboratory testing, and data collection. XW, JY, WL, and MW coordinated the data collection. All authors have read and agreed to the published version of the manuscript.

## Footnotes

1
www.crd.york.ac.uk/PROSPERO
