# Supplementary material for: Circulating saturated fatty acids and risk of gestational diabetes mellitus: A cross-sectional study and meta-analysis
Source: Front Nutr. 2022 Aug 1;9:903689. doi: 10.3389/fnut.2022.903689 (PMC9376316; doi:10.3389/fnut.2022.903689)
Supplement: Supplementary file 1 [file Data_Sheet_1.docx]

Supplementary Material

# Supplementary Text

## Supplementary Text 1. PICO strategy

### Participants/population.

Gestational diabetes mellitus patients according to the Carpenter–Coustan criteria (C&C), International Association of Diabetes and Pregnancy Study Groups (IADPSG) criteria, National Diabetes Data Group (NDDG) criteria, WHO criteria, and International Classification of Diseases codes and local guidelines or criteria

### Intervention(s), exposure(s).

Circulating saturated fatty acids will be used as the exposure of interest. Inclusion: the concentration or proportion of circulating saturated fatty acids were reported, such as myristic acid, palmitic acid, stearic acid, arachidic acid, behenic acid, lignoceric acid.

### Comparator(s)/control.

(1) For the study of saturated fatty acid concentrations or percentages as continuous variables, pregnant women without gestational diabetes mellitus served as controls

(2) For prospective studies reporting saturated fatty acid concentrations or percentages as categorical variables, the low exposure group served as a control

### Main outcome(s).

The circulating saturated fatty acids profiles measured by GC or GC/MS method in healthy and GDM women, such as the concentration (or percentage) of myristic acid, palmitic acid, stearic acid, arachidic acid, behenic acid, lignoceric acid.

### Measures of effect

SMD

### Additional outcome(s).

Incidence of gestational diabetes mellitus

### Measures of effect

Odds ratios (OR) estimates with 95% CI or statistics which can be converted to the required format.

## Supplementary Text 2. Electronic search strategy (until October 2021)

### PubMed

#1 ("diabetes pregnancy induced"[Title/Abstract] OR "diabetes pregnancy induced"[Title/Abstract] OR "pregnancy induced diabetes"[Title/Abstract] OR "gestational diabetes"[Title/Abstract] OR "diabetes mellitus gestational"[Title/Abstract] OR "gestational diabetes mellitus"[Title/Abstract] OR "diabetes, gestational"[MeSH Terms])

#2 ("Fatty Acids"[MeSH Terms] OR "Fatty Acid"[Title/Abstract] OR "Saturated Fatty Acids"[Title/Abstract] OR " Saturated Fatty Acid"[Title/Abstract] OR " Fatty Acids, Saturated"[Title/Abstract] OR " Acid, Saturated Fatty"[Title/Abstract])

#1 AND #2

### Web of science

#1 ((((((TS= (Diabetes, Gestational)) OR TS= (gestational diabetes mellitus)) OR TS= (diabetes mellitus gestational)) OR TS= (gestational diabetes)) OR TS= (pregnancy induced diabetes)) OR TS= (diabetes pregnancy induced)) OR TS= (diabetes pregnancy induced)

#2 (((((TS= (Fatty Acids)) OR TS= (Fatty Acid)) OR TS= (Saturated Fatty Acids)) OR TS= (Saturated Fatty Acid)) OR TS= (Fatty Acids, Saturated)) OR TS= (Acid, Saturated Fatty)

(#1 AND #2) NOT (DT==("REVIEW"))

### Scopus

#1 TITLE-ABS-KEY (fatty AND acid) #2 TITLE-ABS-KEY (gestational AND diabetes AND mellitus) #3 TITLE-ABS-KEY (review) (#1 AND #2) NOT #3

### CNKI

#1 ((TS= (gestational diabetes mellitus)) OR TI= (gestational diabetes mellitus)) OR AB= (gestational diabetes mellitus) (in Chinese)

#2 ((TS= (fatty acid)) OR TI= (fatty acid)) OR AB= (fatty acid) (in Chinese)

#1 AND #2

### CMB

#1 Common words= (gestational diabetes mellitus) (in Chinese)

#2 (Common words= (fatty acid) OR Common words= (Saturated Fatty Acid)) (in Chinese)

#1 AND #2

# Supplementary Tables

## Supplementary Table 1. Plasma SFAs (concentration, mg/L) in pregnant women with versus without GDM in the second trimester.

|  | **Non-GDM** | **GDM** | **Total** | ***p* value** ^*^ |
| --- | --- | --- | --- | --- |
|  | **Median (P25, P75)** | **Median (P25, P75)** | **Median (P25, P75)** |  |
| Myristic acid (C14:0) | 33.1 (22.2, 58.7) | 28.6 (22.1, 43.4) | 32.6 (22.2, 55.8) | 0.026 |
| Palmitic acid (C16:0) | 973 (799, 1190) | 976 (856, 1250) | 973 (805, 1200) | 0.503 |
| Stearic acid (C18:0) | 210 (168, 270) | 166 (134, 205) | 206 (163, 264) | <0.001 |
| Arachidic acid (C20:0) | 2.84 (1.43, 5.48) | 1.64 (1.16, 2.24) | 2.63 (1.37, 5.27) | <0.001 |
| Behenic acid (C22:0) | 5.70 (0.738, 11.6) | 0.738 (0.738, 1.52) | 4.90 (0.738, 11.1) | <0.001 |
| Lignoceric acid (C24:0) | 2.38 (2.38, 4.56) | 2.38 (2.38, 2.38) | 2.38 (2.38, 4.39) | <0.001 |
| VLcSFAs | 21.0 (8.84, 74.7) | 7.98 (7.41, 9.21) | 18.7 (8.37, 71.6) | <0.001 |
| LcSFAs | 1270 (1040, 1610) | 1170 (1030, 1510) | 1260 (1040, 1590) | 0.224 |
| Total SFAs | 1470 (1200, 1850) | 1410 (1190, 1780) | 1460 (1200, 1850) | 0.419 |
| Total FAs | 3670 (3120, 4320) | 3460 (2960, 4000) | 3660 (3110, 4310) | 0.048 |

* The data of GDM and non-GDM pregnant women were compared.

## Supplementary Table 2. Characteristics of included studies that evaluated SFAs in women with and without GDM.

| **Study** | **Study type** | **Age (mean), year** | **GDM Pre-BMI** | **Country** | **Sampling type** | **Measurement method** | **Sampling time** | **Case ascertainment** | **Case ascertainment time** | **No. of participant** | **No. of patient** | **No. of control** |
| --- | --- | --- | --- | --- | --- | --- | --- | --- | --- | --- | --- | --- |
| Wijendran, V., et al. 1999 (g-i) (1) | case-control | 30.7 | Overweight/Obese | US | Plasma | GC | (g) GWs 27–30 (h) GWs 33–35 (i) GWs 36–39 | the O'Sullivan and Mahan criteria and the NDDG criteria | GWs 24–30 | 30 | 15 | 15 |
| Wijendran, V., et al. 2000 (2) | case-control | 31.5 | Lean | US | RBC | GC | GWs 36–39 | the O'Sullivan and Mahan criteria | GWs 24–30 | 25 | 13 | 12 |
| Min, Y., et al. 2004 (a-c) (3) | case-control | 29.4 | Overweight/Obese | UK | (a) Plasma PC (b) RBC PC (c) RBC PE | GC | GWs 28-34 | EASD criteria 1979 | GWs 28-34 | 114 | 53 | 61 |
| Thomas, B., et al. 2004 (a-c) (4) | case-control | 29.6 | Overweight/Obese | UK | (a) Plasma PC (b) Plasma TG (c) Plasma CE | GC | GWs 28-34 | EASD criteria 1979 | GWs 28-34 | 88 | 44 | 44 |
| Min, Y., et al. 2005 (a-d) (5) | case-control | 30.5 | Overweight/Obese | UK | (a) Plasma TG (b) Plasma PC (c) RBC PC (d) RBC PE | GC | at delivery | EASD criteria 1979 | GWs 28-34 | 58 | 34 | 24 |
| Min, Y., et al. 2006 (a-f) (6) | case-control | 29.9 | Lean | South Korea | (a) Plasma TG (b) Plasma PC (c) RBC PC (d) RBC PE (e) Plasma SM (f) RBC SM | GC | before delivery | NDDG criteria | GWs 24-30 | 24 | 12 | 12 |
| Ortega-Senovilla, H., et al. 2009 (7) | case-control | 34.7 | — | Italy | Plasma | GC | at delivery | CC criteria | GWs 24-28 | 45 | 15 | 30 |
| Chen, X., et al. 2010 (8) | nested case-control | 22.7 | Overweight/Obese | US | Serum | GC-MS | GWs 16.5±0.16 | CC criteria | GWs 27.7±0.2 | 147 | 49 | 98 |
| Zhao, J. P., et al. 2014 (9); Zhao, J. P., et al. 2016 (g-h) (10) | nested case-control | 31.3 | Overweight/Obese | Canada | Plasma | GC | (g) GWs 24–28 (h) GWs 32–35 | ADA diabetes diagnostic criteria | GWs 24-28 | 108 | 24 | 84 |
| Yi K. 2015 (11) | case-control | 28.9 | Lean | China | RBC | GC | ＞ GWs 28 | IADPSG criteria | GWs 24-28 | 60 | 30 | 30 |
| Burlina, S., et al. 2017 (12) | case-control | 33.6 | Lean | Italy | Plasma | GC | 3rd trimester | CC criteria | GWs 24-28 | 42 | 21 | 21 |
| Zhu, Y., et al. 2018 (g-h) (13) | nested case-control | 30.4 | Overweight/Obese | US | Plasma | GC | (g) GWs 10-14 (h) GWs 15-26 | CC criteria | GWs 10–14 and 15–26 | 321 | 107 | 214 |
| Bukowiecka-Matusiak, M., et al. 2018 (14) | case-control | 30.5 | Lean | Poland | RBC | GC | GWs 24–28 | WHO criteria 2013 | GWs 24-28 | 43 | 32 | 11 |
| Li, P. 2019 (15) | case-control | 29.8 | Lean | China | Plasma | GC-MS | GWs 26-30 | ADA diabetes diagnostic criteria | ＞GWs 24 | 434 | 217 | 217 |
| Prieto-Sánchez, M. T., et al. 2019 (g-h, j-k) (16) | case-control | 32.9 | Lean or Overweight/Obese | Spain | Serum | GC | (g) 3rd trimester (h) at delivery | NDDG criteria, (j) Lean-GDM (k) Obese-GDM | GWs 24-28 | 68 | 43 | 25 |
| Ortega-Senovilla, H., et al. 2020 (j-k) (17) | case-control | 30.6 | Overweight/Obese | Germany | Serum | GC | at delivery | CC criteria: (j) GDM without insulin (k) GDM with insulin | GWs 26 | 264 | 84 | 180 |
| Huang, Y., et al. 2020 (18); Li, X., et al. 2020 (19); Pan, X. F., et al. 2021 (k) (20) | nested case-control | 30.3 | Lean | China | Plasma | GC-MS | GWs 13±1 | IADPSG 2011 | GWs 24-28 | 610 | 305 | 305 |
| Pan, X. F., et al. 2021 (j) (20) | nested case-control | 27.8 | Lean | China | Plasma | GC-MS | GWs 10.3±2 | IADPSG 2010 | GWs 24-28 | 1008 | 336 | 672 |
| Zhang, T., et al. 2021 (g-i, j-l) (21) | cohort | 28.0 | Lean | China | Plasma | GC-MS | (g) GWs 11-14 (h) GWs 22-28 (i) GWs 32-34 | IADPSG thresholds (j) FPG (k) 1h/2h-PG (l) FPG&1h/2h-PG | GWs 24-28 | 680 | 195 | 485 |
| Gázquez, A., et al. 2021 (22) | cohort | 33.2 | Lean | Spain | Serum | GC | 2nd trimester | NDDG criteria | GWs 24 | 635 | 49 | 586 |
| Wang, H., et al. 2021 (23) | nested case-control | 29.2 | Lean | China | Serum | GC-MS | 1st trimester | IADPSG criteria | GWs 24-28 | 486 | 243 | 243 |
| Tryggvadottir E. 2021 (24) | cohort | 30.3 | Overweight/Obese | Iceland | Plasma | GC | GWs 11-14 | IADPSG criteria | GWs 24-28 | 853 | 127 | 726 |
| Li, L., et al. 2021 (g-h) | nested case-control | 28.0 | Overweight/Obese | US | Plasma | GC | (g) GWs 23-31 (h) GWs 33-39 | CC criteria | GW 10-14 and 15-26 and 23-31 | 170 | 85 | 85 |
| Raczkowsk, B.A.,et al.2021 (g-h, j-k) (25) | cohort | 23.5 | Lean | Poland | Serum | GC-MS | (g) GWs 8-14 (h) GWs 24-28 | (j) aGT-GDM (k) aFPG-GDM | GWs 24-28 | 662 | 99 | 563 |
| current original study | cross-sectional | 30,6 | Lean | China | Plasma | GC | GWs 24-28 | IADPSG criteria | GWs 24-28 | 807 | 746 | 61 |
| TG: triacylglycerols; PC: phosphatidylcholine; RBC: red blood cell; PE: phosphatidylethanolamine; SM: sphingomyelin; GC: gas chromatography; GC-MS: gas chromatography spectrometry; GW: Gestational week; CE: cholesterol esters. EASD: The European Association for the Study of Diabetes; ADA: American Diabetes Association; NDDG: the National Diabetes Data Group; IADPSG: International Association of Diabetes and Pregnancy Study Groups; CC: Carpenter–Coustan (American College of Obstetrics and Gynecologists primary recommendation); aGT: abnormal glucose tolerance; aFPG: abnormal fasting plasma glucose. | | | | | | | | | | | | |

## Supplementary Table 3. Characteristics of studies that evaluated the associations between SFAs and incidence of GDM.

| **Study** | **Study name** | **Types of saturated fatty acids** | **Adjusted covariates** | **Fatty acid’s categories** |
| --- | --- | --- | --- | --- |
| Zhu, Y., et al. 2018 (g-h) (13) | the National Institute of Child Health and Human Development Fetal Growth Studies–Singleton Cohort | Myristic acid, palmitic acid, stearic acid | Age, the gestational-mass week at blood collection, parity, family history of diabetes, and pre-pregnancy BMI | Q1-Q4 |
| Huang, Y., et al. 2020; Li, X., et al. 2020; Pan, X. F., et al. 2021 (k) (18-20) | (k) longitudinal birth cohort project conducted at Wuhan Medical & Healthcare Center for Women and Children (MHCWC) study | Total SFAs, myristic acid, palmitic acid, stearic acid, arachidic acid, behenic acid, lignoceric acid | Age, parity, education level, pre-pregnancy BMI, and passive smoking | Q1-Q3 |
| Pan, X. F., et al. 2021 (j) (20) | (j) Tongji-Shuangliu Birth Cohort (TSBC) study | Myristic acid, palmitic acid, stearic acid, arachidic acid, behenic acid, lignoceric acid | Age, parity, education level, pre-pregnancy body mass index, and passive smoking | Q1-Q4 |
| Zhang, T., et al. 2021 (g-i, j-l) (21) | the Complex Lipids in Mothers and Babies (CLIMB) study | Myristic acid, palmitic acid, stearic acid, arachidic acid, behenic acid, lignoceric acid | Maternal age, education, gestational age, parity, cigarette smoking, alcohol drinking, physical activity, pre-pregnancy BMI, family history of diabetes, and history of GDM | Q1-Q3 |
| Li, P. 2019 (15) | — | Total SFAs, myristic acid, palmitic acid, stearic acid, lignoceric acid | Maternal age, gestational age, pre-pregnancy BMI, parity, cigarette smoking, alcohol drinking, family history of diabetes | Q1-Q4 |
| Yi K. 2015 (11) | — | Total SFAs, myristic acid, palmitic acid, stearic acid | Pre-pregnancy BMI | Q1-Q3 |

## Supplementary Table 4. Subgroups Analysis of SFA levels in pregnant women with and without GDM.

|  | **Myristic acid (C14:0)** | | | |  | **Palmitic acid (C16:0)** | | | |  | **Stearic acid (C18:0)** | | | |
| --- | --- | --- | --- | --- | --- | --- | --- | --- | --- | --- | --- | --- | --- | --- |
| **Subgroup Factors** | **n** | **SMD (95%CI)** | **I^2^, %** | ***p*** |  | **n** | **SMD (95%CI)** | **I^2^, %** | ***p*** |  | **n** | **SMD (95%CI)** | **I^2^, %** | ***p*** |
| **Percentage** |  |  |  |  |  |  |  |  |  |  |  |  |  |  |
| **Sample type** |  |  |  |  |  |  |  |  |  |  |  |  |  |  |
| Plasma/Serum | 9 | 0.006 [-0.164; 0.176] | 81 | 0.941 |  | 12 | 0.267 [ 0.151; 0.382] | 60 | **<0.001** |  | 12 | -0.108 [ -0.270; 0.053] | 80 | 0.187 |
| RBC | 2 | -0.356 (-0.651; -0.061) | 0 | **0.018** |  | 3 | 0.208 [ -0.920; 1.336] | 91 | 0.718 |  | 3 | 0.101 [ -0.871; 1.073] | 87 | 0.839 |
| **Prepregnant-BMI of GDM** | |  |  |  |  |  |  |  |  |  |  |  |  |  |
| Lean | 7 | 0.034 [-0.178; 0.246] | 81 | 0.752 |  | 10 | 0.337 [ 0.132; 0.542] | 81 | **0.001** |  | 10 | 0.003 [-0.278; 0.285] | 90 | 0.983 |
| Overweight/Obese | 4 | -0.079 [-0.331; 0.174] | 77 | 0.541 |  | 5 | 0.216 [ 0.044; 0.388] | 54 | **0.014** |  | 5 | -0.128 [-0.235; -0.020] | 29 | **0.020** |
| **Concentration** |  |  |  |  |  |  |  |  |  |  |  |  |  |  |
| **Sampling type** |  |  |  |  |  |  |  |  |  |  |  |  |  |  |
| Plasma/Serum | 7 | 0.187 [ 0.055; 0.320] | 69 | **0.006** |  | 9 | 0.059 [-0.082; 0.200] | 76 | **<0.001** |  | 8 | -0.017 [-0.162; 0.128] | 77 | 0.817 |
| RBC | 0 | — | — | — |  | 1 | 0.331 [-0.460; 1.122] | — | 0.779 |  | 1 | 0.279 (-0.510; 1.068) | — | 0.489 |
| **Prepregnant-BMI of GDM** | |  |  |  |  |  |  |  |  |  |  |  |  |  |
| Lean | 4 | 0.169 [ 0.002; 0.336] | 76 | **0.047** |  | 5 | 0.141 [ 0.063; 0.220] | 47 | **<0.001** |  | 4 | 0.038 [-0.125; 0.201] | 73 | 0.647 |
| Overweight/Obese | 3 | 0.184 [ 0.044; 0.325] | 40 | **0.010** |  | 5 | -0.049 [-0.390; 0.293] | 86 | 0.779 |  | 5 | -0.090 [-0.371; 0.192] | 79 | 0.532 |

| **Supplementary Table 4. *(continued)*** | | | | | | | | | | | | | | |
| --- | --- | --- | --- | --- | --- | --- | --- | --- | --- | --- | --- | --- | --- | --- |
|  | **Arachidic acid (C20:0)** | | | |  | **Behenic acid (C22:0)** | | | |  | **Lignoceric acid (C24:0)** | | | |
| **Subgroup Factors** | **n** | **SMD (95%CI)** | **I^2^, %** | ***p*** |  | **n** | **SMD (95%CI)** | **I^2^, %** | ***p*** |  | **n** | **SMD (95%CI)** | **I^2^, %** | ***p*** |
| **Percentage** |  |  |  |  |  |  |  |  |  |  |  |  |  |  |
| **Sample type** |  |  |  |  |  |  |  |  |  |  |  |  |  |  |
| Plasma or serum | 6 | -0.144 [-0.228; -0.061] | 43 | **<0.001** |  | 4 | -0.229 [-0.566; 0.109] | 92 | 0.184 |  | 5 | -0.123 [-0.408; 0.162] | 91 | 0.397 |
| RBC | 1 | 0.376 [ 0.049; 0.702] | - | **0.024** |  | 1 | -0.085 [-0.409; 0.238] | 0 | 0.606 |  | 1 | -0.353 [-0.679; -0.027] | 0 | **0.034** |
| **Prepregnant-BMI of GDM** |  |  |  |  |  |  |  |  |  |  |  |  |  |  |
| Lean | 4 | -0.081 [-0.327; 0.165] | 83 | 0.518 |  | 4 | -0.290 [-0.695; 0.116] | 94 | 0.161 |  | 5 | -0.231 [-0.557; 0.095] | 93 | 0.164 |
| Overweight/Obese | 3 | -0.171 [-0.346; 0.005] | 0 | 0.056 |  | 1 | -0.030 [-0.242; 0.183] | 0 | 0.784 |  | 1 | 0.058 [-0.154; 0.271] | 14 | 0.591 |
| **Concentration** |  |  |  |  |  |  |  |  |  |  |  |  |  |  |
| **Sampling type** |  |  |  |  |  |  |  |  |  |  |  |  |  |  |
| Plasma or serum | 5 | -0.069 [-0.186; 0.048] | 51 | 0.248 |  | 2 | -0.217 (-0.389; -0.045) | 71 | **0.014** |  | 2 | -0.208 (-0.350; -0.067) | 58 | **0.004** |
| RBC | 0 | — | — | — |  | 0 | — | — | — |  | 0 | — | — | — |
| **GDM Pre-BMI** |  |  |  |  |  |  |  |  |  |  |  |  |  |  |
| Lean | 3 | -0.012 [-0.130; 0.105] | 50 | 0.835 |  | 2 | -0.217 (-0.389; -0.045) | 71 | **0.014** |  | 2 | -0.208 [-0.350; -0.067] | 58 | **0.004** |
| Overweight/Obese | 2 | -0.385 [-0.636; -0.135] | 0 | **0.003** |  | 0 | — | — | — |  | 0 | — | — | — |
| Early-pregnancy: GWs 1-12; Mid-pregnancy: GWs 13-28; Late-pregnancy: GWs 28-40. Prepregnant-BMI was divided into two categories according to the BMI classification standard of WHO: Lean <25.0kg/m^2^, Overweight/Obese≥25.0kg/m^2^. | | | | | | | | | | | | | | |

## Supplementary Table 5. NOS score of included studies.

| **Study** | **Selection** | **Comparability** | **Exposure** | **Overall** |
| --- | --- | --- | --- | --- |
| Wijendran, V., et al. 1999 (g-i) | 4 | 1 | 3 | 8 |
| Wijendran, V., et al. 2000 | 3 | 0 | 3 | 6 |
| Min, Y., et al. 2004 (a-c) | 4 | 0 | 3 | 7 |
| Thomas, B., et al. 2004 (a-c) | 4 | 0 | 3 | 7 |
| Min, Y., et al. 2005 (a-d) | 3 | 2 | 3 | 8 |
| Min, Y., et al. 2006 (a-f) | 4 | 1 | 3 | 8 |
| Ortega-Senovilla, H., et al. 2009 | 4 | 0 | 3 | 7 |
| Chen, X., et al. 2010 (g-h) | 4 | 2 | 3 | 9 |
| Zhao, J. P., et al. 2014 | 3 | 1 | 3 | 7 |
| Yi K. 2015 | 3 | 0 | 3 | 6 |
| Zhao, J. P., et al. 2016 (g-h) | 4 | 2 | 3 | 9 |
| Burlina, S., et al. 2017 | 3 | 0 | 3 | 6 |
| Zhu, Y., et al. 2018 (g-h) | 4 | 2 | 3 | 9 |
| Bukowiecka-Matusiak, M., et al. 2018 | 4 | 0 | 3 | 7 |
| Prieto-Sánchez, M. T., et al. 2019 (g-h, j-k) | 3 | 1 | 3 | 7 |
| Zhu, Y., et al. 2019 (g-h) | 4 | 2 | 3 | 9 |
| Li, P. 2019 | 3 | 2 | 3 | 8 |
| Ortega-Senovilla, H., et al. 2020 (j-k) | 4 | 2 | 3 | 9 |
| Huang, Y., et al. 2020 | 4 | 2 | 3 | 9 |
| Li, X., et al. 2020 | 4 | 2 | 3 | 9 |
| Pan, X. F., et al. 2021 (j-k) | 4 | 2 | 3 | 9 |
| Zhang, T., et al. 2021 (g-i, j-l) | 4 | 2 | 3 | 9 |
| He, X. J., et al. 2021 | 4 | 0 | 3 | 7 |
| Gázquez, A., et al. 2021 | 4 | 2 | 3 | 9 |
| Wang, H., et al. 2021 | 3 | 2 | 3 | 8 |
| Tryggvadottir E. 2021 | 3 | 2 | 3 | 8 |
| Li, L., et al. 2021 (g-h) | 4 | 2 | 3 | 9 |
| Raczkowsk, B.A.,et al.2021 (g-h, j-k) | 3 | 2 | 3 | 8 |

# Supplementary Figures

Literature retrieved from database (n=2579)

- PubMed: 429
- Web of Science: 1142
- Scopus: 462
- CNKI: 207
- CMB: 339

Others (n=2)

Remove duplications (n=956)

Literature screened by title and abstract (n=1625)

Exclusion of irrelevant literature (n=1593)

Literature screened after full text browsing (n=36)

- Population not meeting inclusion criteria (n=4)
- No data available (n=4)
- Duplicate publication (n=5)
- Printing error (n=1)

Final included literature (n=22)

**Screening**

**Identification**

**Eligibility**

**Included**

Final included literature (n=25)

- Current original study (n=1)
- Update to March 2022 (n=2)

## Supplementary Figure 1. Flow diagram of the literature research for meta- analysis.

##
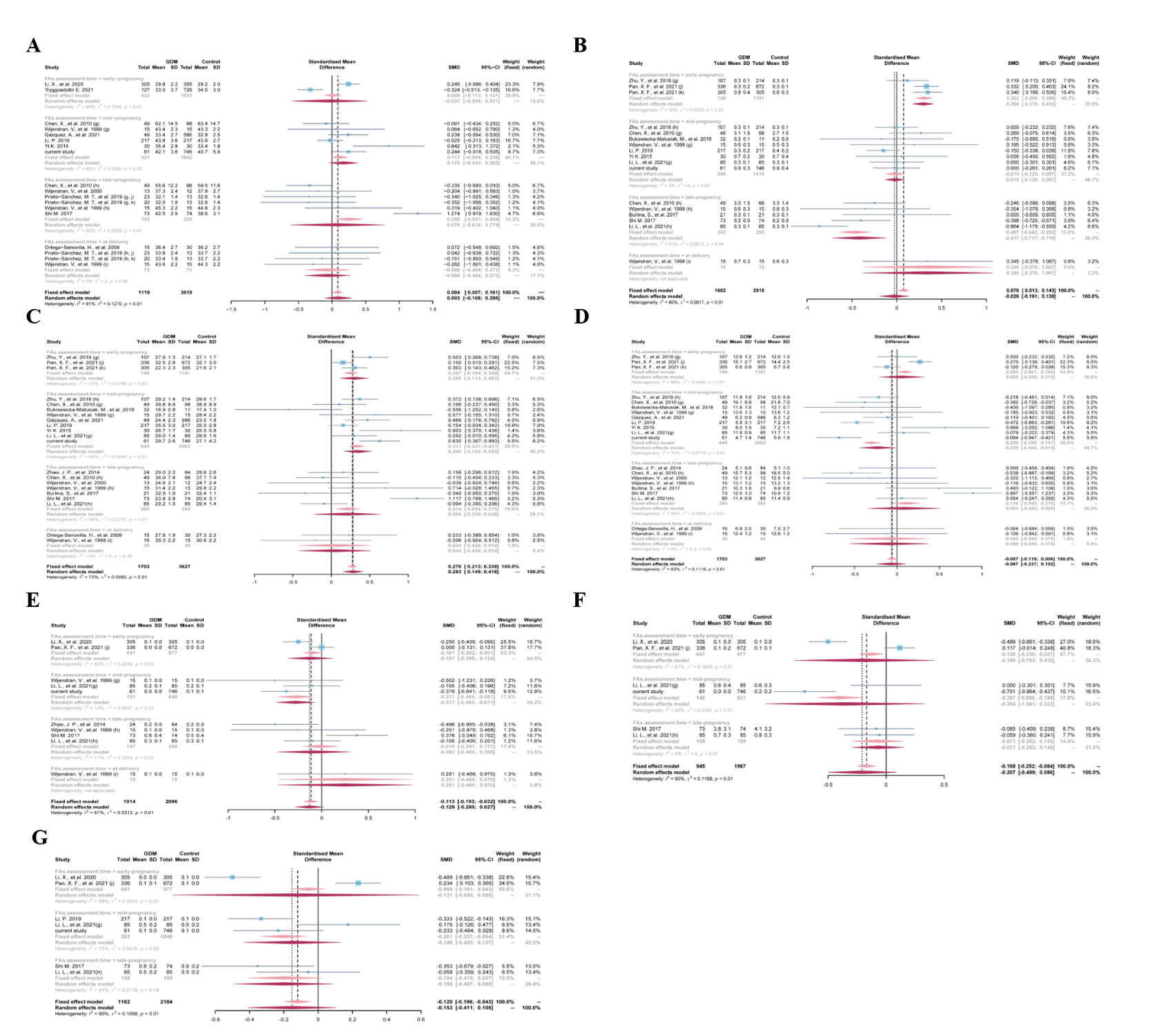
Supplementary Figure 2. Meta-analysis of SMDs for total SFAs or each SFA (% of total fatty acids) in pregnant women with and without GDM. (A) Total SFAs; (B) Myristic acid; (C) Palmitic acid; (D) Stearic acid; (E) Arachidic acid; (F) Behenic acid; (G) Lignoceric acid. Both fixed - and random effects models were presented in this figure, but specific models were selected for reporting based on heterogeneity in the main text (Figure 3). The specific meaning of the (a-l) marked after the study name was listed in supplementary Table 2. The (a-f) markers represented FAs measurements with different sampling types in the same study, the (g-i) markers represented FAs measurements with different time in the same study, and the (j-l) markers represented FAs measurements with different GDM diagnostic subgroups in the same study.

##
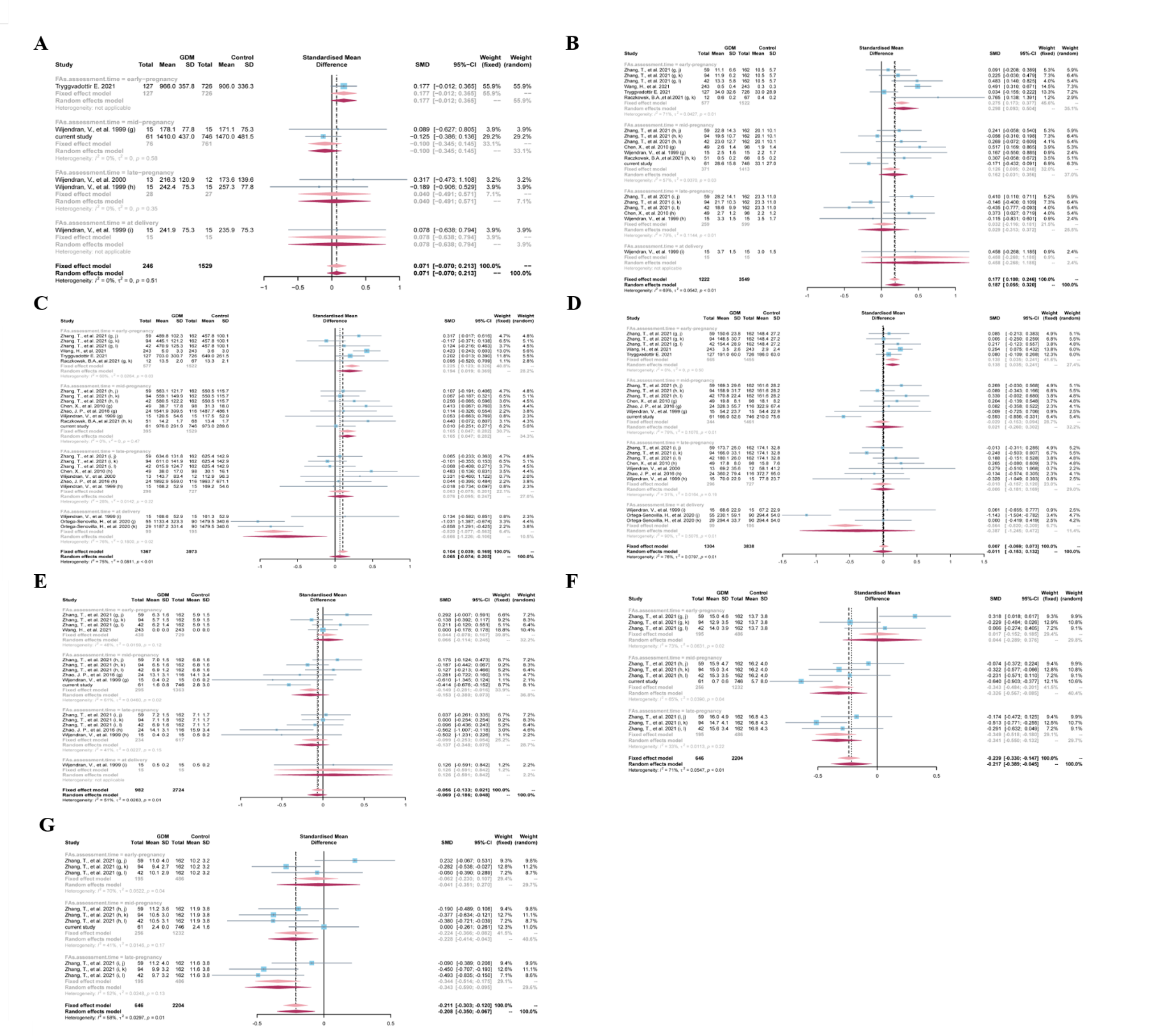
Supplementary Figure 3. Meta-analysis of SMDs for total SFAs or each SFA (concentration, mg/L) in pregnant women with and without GDM. (A) Total SFAs; (B) Myristic acid; (C) Palmitic acid; (D) Stearic acid; (E) Arachidic acid; (F) Behenic acid; (G) Lignoceric acid. Subgroup analysis was performed according to the sampling time of biological samples. Both fixed - and random effects models were presented in this figure, but specific models were selected for reporting based on heterogeneity in the main text (Figure 4). The specific meaning of the (a-l) marked after the study name was listed in supplementary Table 2. The (a-f) markers represented FAs measurements with different sampling types in the same study, the (g-i) markers represented FAs measurements with different time in the same study, and the (j-l) markers represented FAs measurements with different GDM diagnostic subgroups in the same study.

##
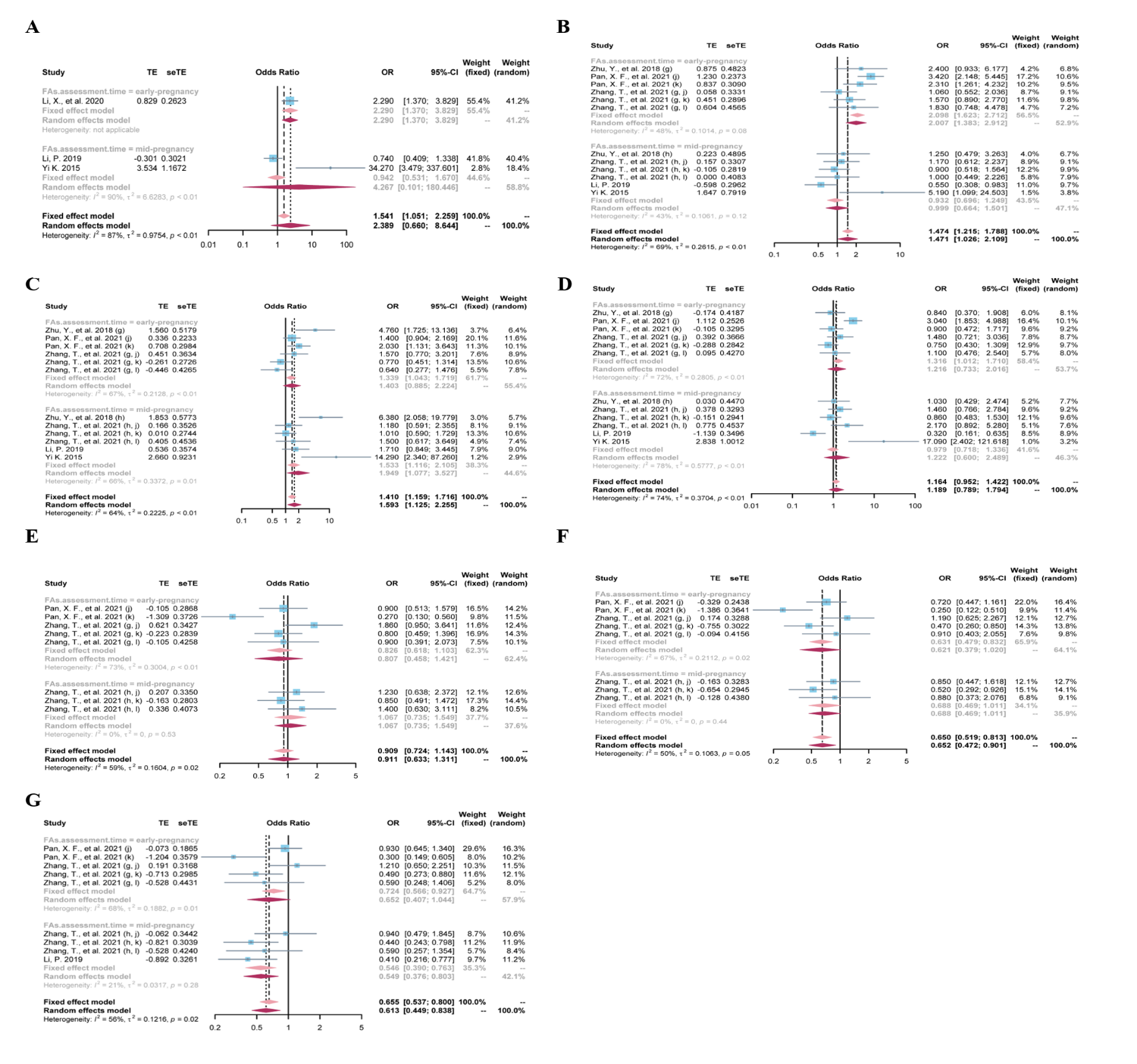
Supplementary Figure 4. Meta-analysis of associations between SFAs and the prevalence of GDM. (A) Total SFAs; (B) Myristic acid; (C) Palmitic acid; (D) Stearic acid; (E) Arachidic acid; (F) Behenic acid; (G) Lignoceric acid. Subgroup analysis was performed according to the sampling time of biological samples. Both fixed - and random effects models were presented in this figure, but specific models were selected for reporting based on heterogeneity in the main text (Figure 5). The specific meaning of the (a-l) marked after the study name was listed in supplementary Table 2. The (a-f) markers represented FAs measurements with different sampling types in the same study, the (g-i) markers represented FAs measurements with different time in the same study, and the (j-l) markers represented FAs measurements with different GDM diagnostic subgroups in the same study.


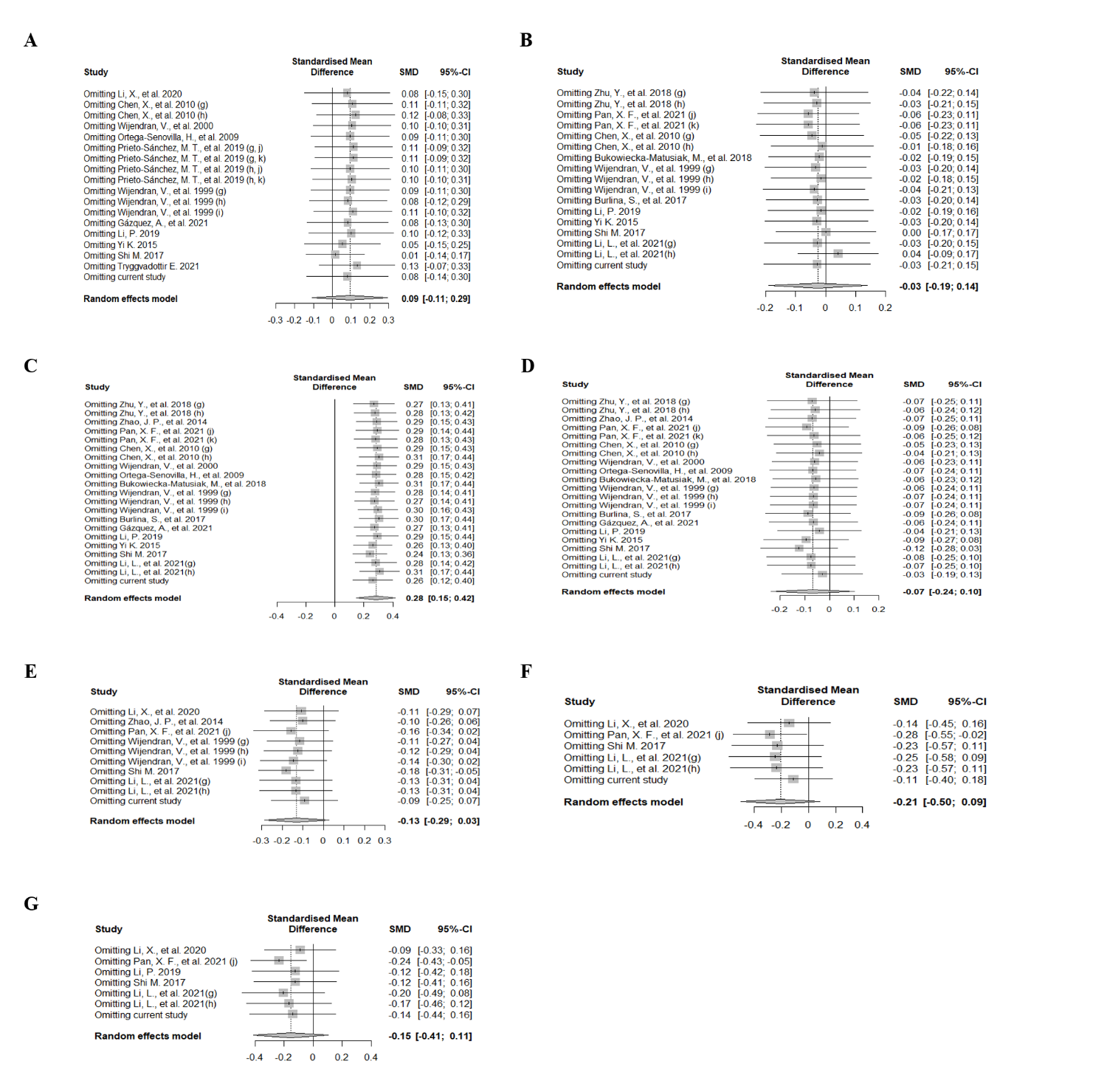


## Supplementary Figure 5. Sensitivity analysis for meta-analysis of SMDs for each SFA or total SFAs (% of total fatty acids) in pregnant women with and without GDM by omitting one study at a time. (A) Total SFAs; (B) Myristic acid; (C) Palmitic acid; (D) Stearic acid; (E) Arachidic acid; (F) Behenic acid; (G) Lignoceric acid. The specific meaning of the (a-l) marked after the study name was listed in supplementary Table 2. The (a-f) markers represented FAs measurements with different sampling types in the same study, the (g-i) markers represented FAs measurements with different time in the same study, and the (j-l) markers represented FAs measurements with different GDM diagnostic subgroups in the same study.

##
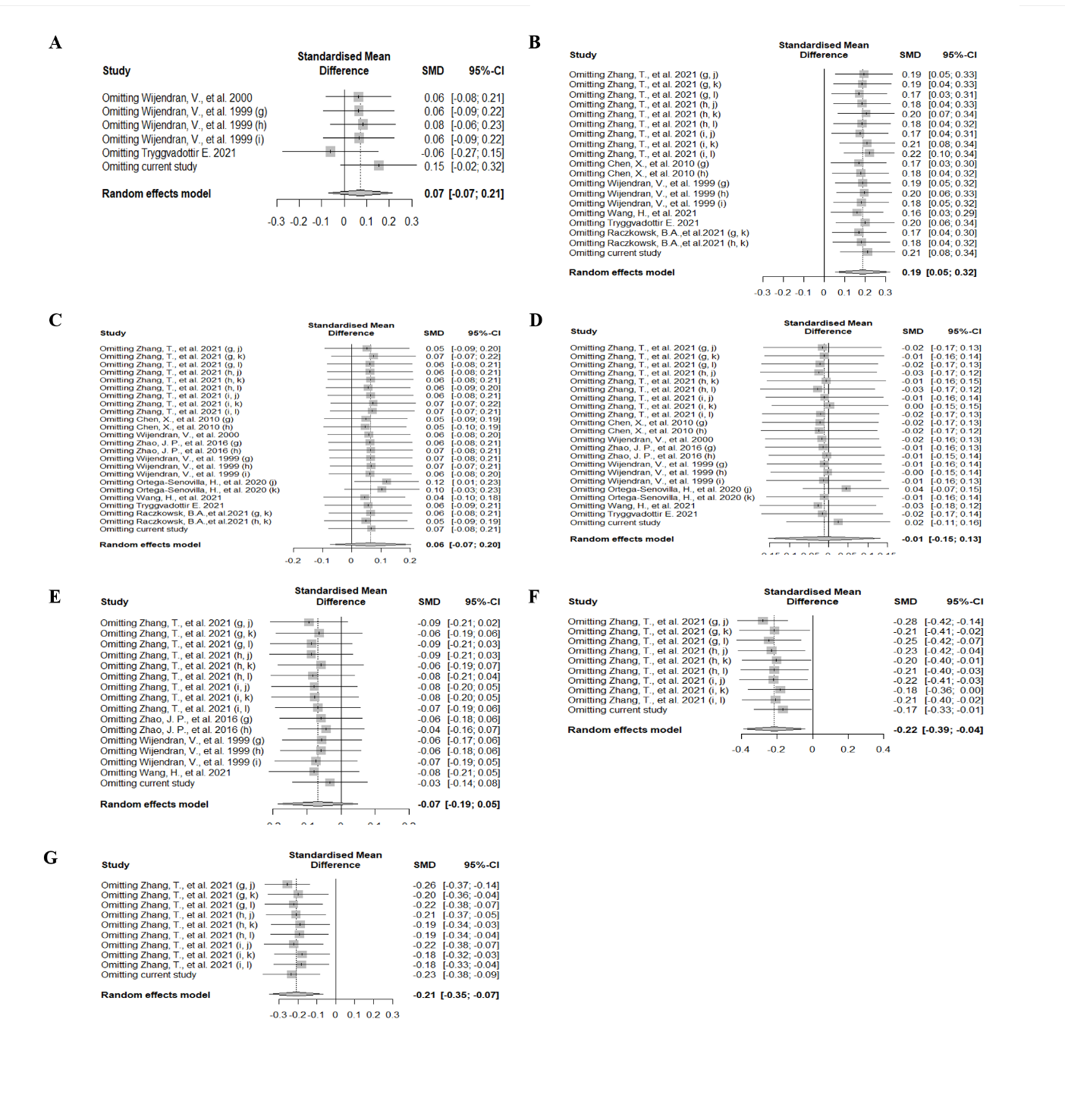
Supplementary Figure 6. Sensitivity analysis for meta-analysis of SMDs for each SFA or total SFAs (concentration, mg/L) in pregnant women with and without GDM by omitting one study at a time. (A) Total SFAs; (B) Myristic acid; (C) Palmitic acid; (D) Stearic acid; (E) Arachidic acid; (F) Behenic acid; (G) Lignoceric acid. The specific meaning of the (a-l) marked after the study name was listed in supplementary Table 2. The (a-f) markers represented FAs measurements with different sampling types in the same study, the (g-i) markers represented FAs measurements with different time in the same study, and the (j-l) markers represented FAs measurements with different GDM diagnostic subgroups in the same study.

##
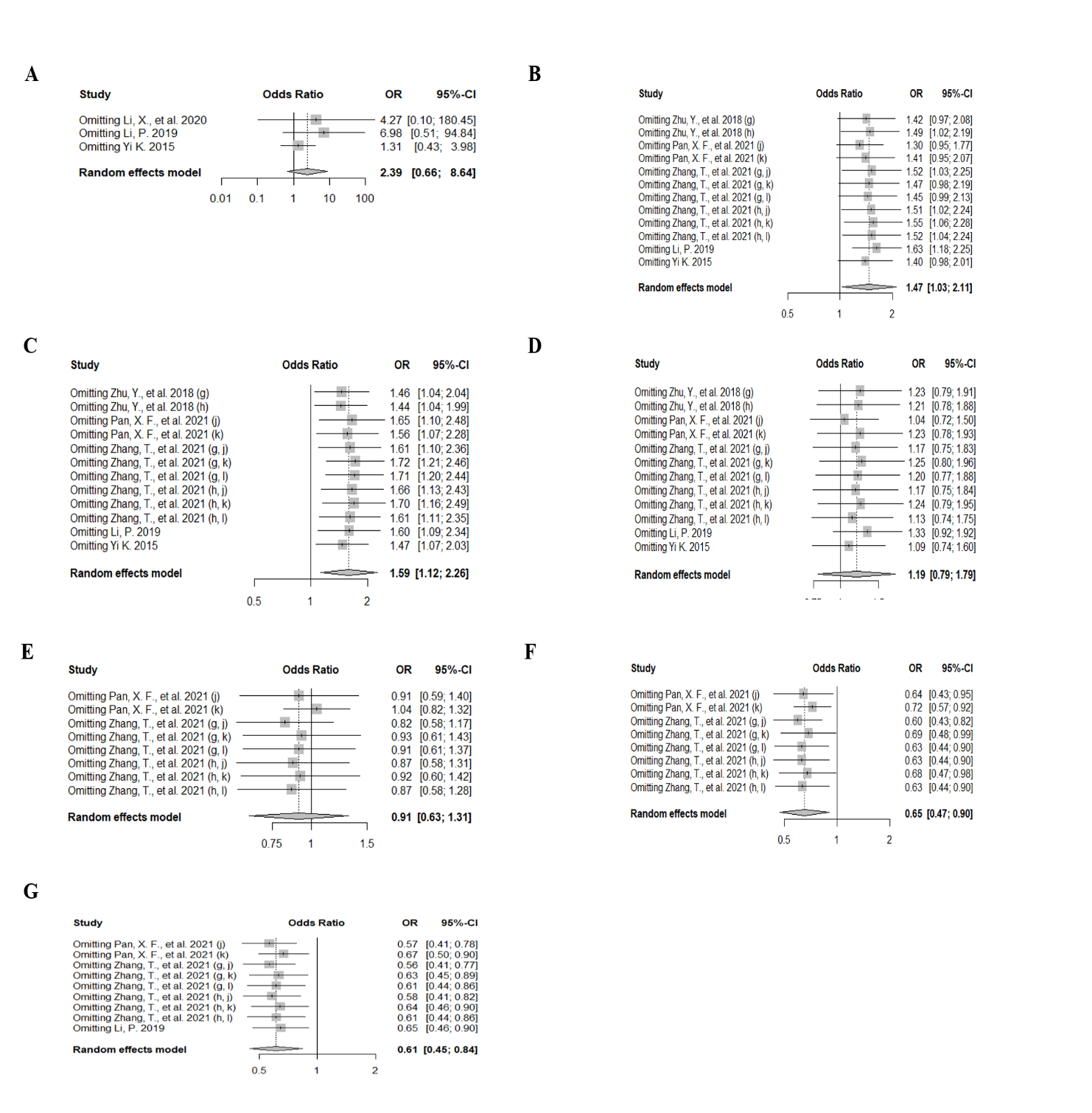
Supplementary Figure 7. Sensitivity analysis for meta-analysis by omitting one study at a time regarding the associations between each SFA (% of total fatty acids) and the incidence of GDM. (A) Total SFAs; (B) Myristic acid; (C) Palmitic acid; (D) Stearic acid; (E) Arachidic acid; (F) Behenic acid; (G) Lignoceric acid. The specific meaning of the (a-l) marked after the study name was listed in supplementary Table 2. The (a-f) markers represented FAs measurements with different sampling types in the same study, the (g-i) markers represented FAs measurements with different time in the same study, and the (j-l) markers represented FAs measurements with different GDM diagnostic subgroups in the same study.


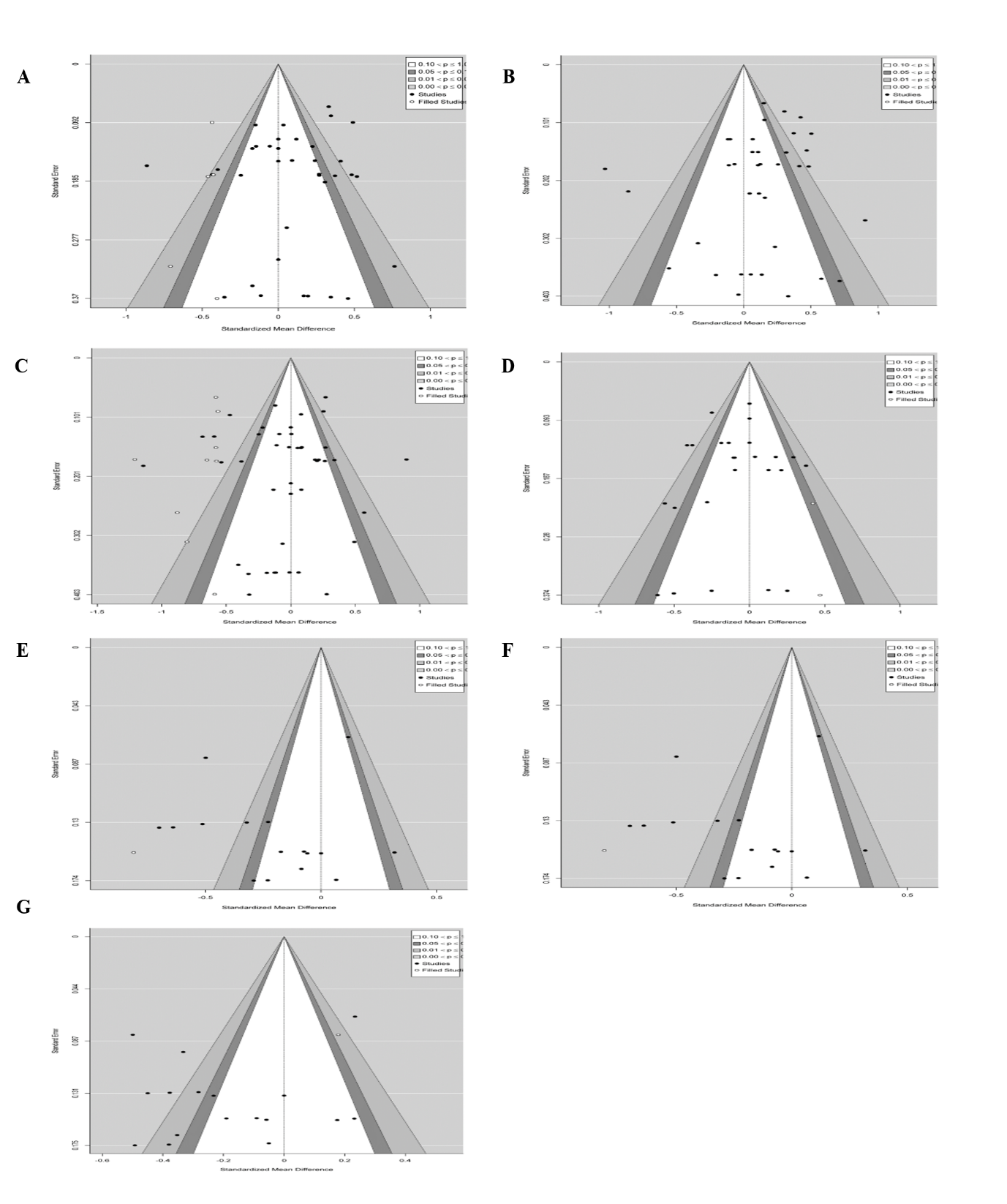


## Supplementary Figure 8. Funnel plot for meta-analysis of SMDs for each SFA or total SFAs. (A) Total SFAs; (B) Myristic acid; (C) Palmitic acid; (D) Stearic acid; (E) Arachidic acid; (F) Behenic acid; (G) Lignoceric acid. Egger’s test was adopted and p = 0.6192 for total SFAs, 0.9704 for myristic acid, 0.3955 for palmitic acid, 0.9135 for stearic acid, 0.2658 for arachidic acid, 0.5875 for behenic acid and 0.8565 for lignoceric acid

##
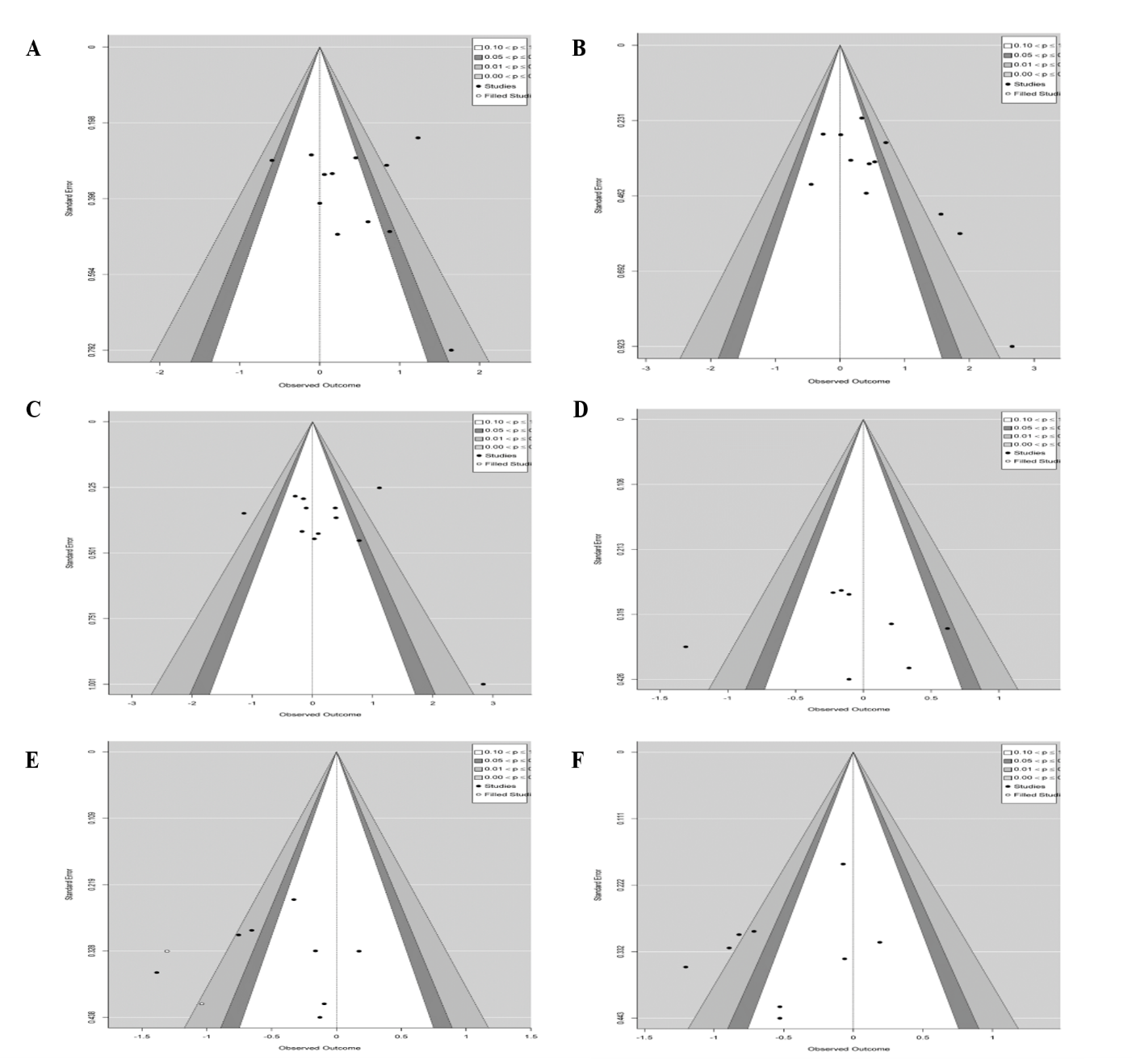
Supplementary Figure 9. Funnel plot for meta-analysis of ORs for each SFA. (A) Myristic acid; (B) Palmitic acid; (C) Stearic acid; (D) Arachidic acid; (E) Behenic acid; (F) Lignoceric acid; (G) too few studies for SFAs. Egger’s test was adopted and *p* = 0.0444 for total SFAs, 0.3256 for myristic acid, 0.0013 for palmitic acid, 0.0554 for stearic acid, 0.9884 for arachidic acid, 0.7710 for behenic acid and 0.3395 for lignoceric acid

# REFERENCES

1. Wijendran V, Bendel RB, Couch SC, Philipson EH, Thomsen K, Zhang X, et al. Maternal Plasma Phospholipid Polyunsaturated Fatty Acids in Pregnancy with and without Gestational Diabetes Mellitus: Relations with Maternal Factors. *The American journal of clinical nutrition* (1999) 70(1):53-61.

2. Wijendran V, Bendel RB, Couch SC, Philipson EH, Cheruku S, Lammi-Keefe CJ. Fetal Erythrocyte Phospholipid Polyunsaturated Fatty Acids Are Altered in Pregnancy Complicated with Gestational Diabetes Mellitus. *Lipids* (2000) 35(8):927-31.

3. Min Y, Ghebremeskel K, Lowy C, Thomas B, Crawford MA. Adverse Effect of Obesity on Red Cell Membrane Arachidonic and Docosahexaenoic Acids in Gestational Diabetes. *Diabetologia* (2004) 47(1):75-81. Epub 2003/11/25. doi: 10.1007/s00125-003-1275-5.

4. Thomas B, Ghebremeskel K, Lowy C, Min Y, Crawford MA. Plasma Aa and Dha Levels Are Not Compromised in Newly Diagnosed Gestational Diabetic Women. *Eur J Clin Nutr* (2004) 58(11):1492-7.

5. Min Y, Lowy C, Ghebremeskel K, Thomas B, Bitsanis D, Crawford MA. Fetal Erythrocyte Membrane Lipids Modification: Preliminary Observation of an Early Sign of Compromised Insulin Sensitivity in Offspring of Gestational Diabetic Women. *Diabetic medicine : a journal of the British Diabetic Association* (2005) 22(7):914-20.

6. Min Y, Nam J-H, Ghebremeskel K, Kim A, Crawford M. A Distinctive Fatty Acid Profile in Circulating Lipids of Korean Gestational Diabetics: A Pilot Study. *Diabetes research and clinical practice* (2006) 73(2):178-83.

7. Ortega-Senovilla H, Alvino G, Taricco E, Cetin I, Herrera E. Gestational Diabetes Mellitus Upsets the Proportion of Fatty Acids in Umbilical Arterial but Not Venous Plasma. *Diabetes care* (2009) 32(1):120-2. doi: 10.2337/dc08-0679.

8. Chen X, Scholl TO, Leskiw M, Savaille J, Stein TP. Differences in Maternal Circulating Fatty Acid Composition and Dietary Fat Intake in Women with Gestational Diabetes Mellitus or Mild Gestational Hyperglycemia. *Diabetes Care* (2010) 33(9):2049-54. Epub 2010/09/02. doi: 10.2337/dc10-0693.

9. Zhao JP, Levy E, Fraser WD, Julien P, Delvin E, Montoudis A, et al. Circulating Docosahexaenoic Acid Levels Are Associated with Fetal Insulin Sensitivity. *PLoS One* (2014) 9(1):e85054. Epub 2014/01/24. doi: 10.1371/journal.pone.0085054.

10. Zhao JP, Levy E, Shatenstein B, Fraser WD, Julien P, Montoudis A, et al. Longitudinal Circulating Concentrations of Long-Chain Polyunsaturated Fatty Acids in the Third Trimester of Pregnancy in Gestational Diabetes. *Diabet Med* (2016) 33(7):939-46. Epub 2015/10/04. doi: 10.1111/dme.12978.

11. Yi K. The Effect of Medical Nutrition Therapy for Gdm on the Composition of Maternal and Neonatal Serum Fatty Acid and Pregnancy Outcome (in Chinese). *dissertation, Qing Dao Uni* (2015).

12. Burlina S, Dalfrà MG, Barison A, Marin R, Ragazzi E, Sartore G, et al. Plasma Phospholipid Fatty Acid Composition and Desaturase Activity in Women with Gestational Diabetes Mellitus before and after Delivery. *Acta diabetologica* (2017) 54(1):45-51. doi: 10.1007/s00592-016-0901-x.

13. Zhu Y, Tsai MY, Sun Q, Hinkle SN, Rawal S, Mendola P, et al. A Prospective and Longitudinal Study of Plasma Phospholipid Saturated Fatty Acid Profile in Relation to Cardiometabolic Biomarkers and the Risk of Gestational Diabetes. *Am J Clin Nutr* (2018) 107(6):1017-26. Epub 2018/06/06. doi: 10.1093/ajcn/nqy051.

14. Bukowiecka-Matusiak M, Burzynska-Pedziwiatr I, Sansone A, Malachowska B, Zurawska-Klis M, Ferreri C, et al. Lipid Profile Changes in Erythrocyte Membranes of Women with Diagnosed Gdm. *PloS one* (2018) 13(9):e0203799. doi: 10.1371/journal.pone.0203799.

15. Li P-Y. Plasma Fatty Acids During Pregnancy and Risk of Gestational Diabetes Mellitus (in Chinese). *dissertation, Huazhong Univ Sci Technol* (2019).

16. Prieto-Sánchez MT, Blanco-Carnero JE, Ruiz-Palacios M, Pagán A, Ruiz-Alcaraz AJ, Larqué E. Increased Alkaline Phosphatase in Cord Blood of Obese Diabetic Mothers Is Associated to Polyunstaurated Fatty Acid Levels. *Annals of nutrition & metabolism* (2019) 75(3):153-62. doi: 10.1159/000504404.

17. Ortega-Senovilla H, Schaefer-Graf U, Herrera E. Pregnant Women with Gestational Diabetes and with Well Controlled Glucose Levels Have Decreased Concentrations of Individual Fatty Acids in Maternal and Cord Serum. *Diabetologia* (2020) 63(4):864-74. doi: 10.1007/s00125-019-05054-x.

18. Huang Y, Li X, Zhang W, Su W, Zhou A, Xu S, et al. Aluminum Exposure and Gestational Diabetes Mellitus: Associations and Potential Mediation by N-6 Polyunsaturated Fatty Acids. *Environmental science & technology* (2020) 54(8):5031-40. doi: 10.1021/acs.est.9b07180.

19. Li X, Huang Y, Xing Y, Hu C, Zhang W, Tang Y, et al. Association of Urinary Cadmium, Circulating Fatty Acids, and Risk of Gestational Diabetes Mellitus: A Nested Case-Control Study in China. *Environ Int* (2020) 137:105527. Epub 2020/02/03. doi: 10.1016/j.envint.2020.105527.

20. Pan X-F, Huang Y, Li X, Wang Y, Ye Y, Chen H, et al. Circulating Fatty Acids and Risk of Gestational Diabetes Mellitus: Prospective Analyses in China. *European journal of endocrinology* (2021) 185(1):87-97. doi: 10.1530/EJE-21-0118.

21. Zhang T, Jiang W-R, Xia Y-Y, Mansell T, Saffery R, Cannon RD, et al. Complex Patterns of Circulating Fatty Acid Levels in Gestational Diabetes Mellitus Subclasses across Pregnancy. *Clinical nutrition (Edinburgh, Scotland)* (2021) 40(6):4140-8. doi: 10.1016/j.clnu.2021.01.046.

22. Gázquez A, Giménez-Bañón MJ, Prieto-Sánchez MT, Martínez-Graciá C, Suárez C, Santaella-Pascual M, et al. Self-Reported Dha Supplementation During Pregnancy and Its Association with Obesity or Gestational Diabetes in Relation to Dha Concentration in Cord and Maternal Plasma: Results from Nela, a Prospective Mother-Offspring Cohort. *Nutrients* (2021) 13(3). doi: 10.3390/nu13030843.

23. Wang H, Yang W, Liu J, Leng J, Li W, Yu Z, et al. Serum Concentrations of Sfas and Cdkal1 Single-Nucleotide Polymorphism Rs7747752 Are Related to an Increased Risk of Gestational Diabetes Mellitus. *The American journal of clinical nutrition* (2021). doi: 10.1093/ajcn/nqab225.

24. Tryggvadottir EA, Gunnarsdottir I, Birgisdottir BE, Hrolfsdottir L, Landberg R, Hreidarsdottir IT, et al. Early Pregnancy Plasma Fatty Acid Profiles of Women Later Diagnosed with Gestational Diabetes. *BMJ open diabetes research & care* (2021) 9(1). doi: 10.1136/bmjdrc-2021-002326.

25. Raczkowska BA, Mojsak P, Rojo D, Telejko B, Paczkowska-Abdulsalam M, Hryniewicka J, et al. Gas Chromatography-Mass Spectroscopy-Based Metabolomics Analysis Reveals Potential Biochemical Markers for Diagnosis of Gestational Diabetes Mellitus. *Front Pharmacol* (2021) 12:770240. Epub 2021/12/07. doi: 10.3389/fphar.2021.770240.
